# Supplementary figures and images for: Unlocking the power of AI for phenotyping fruit morphology in Arabidopsis
Source: Gigascience. 2025 Feb 12;14:giae123. doi: 10.1093/gigascience/giae123 (PMC11816797; doi:10.1093/gigascience/giae123)

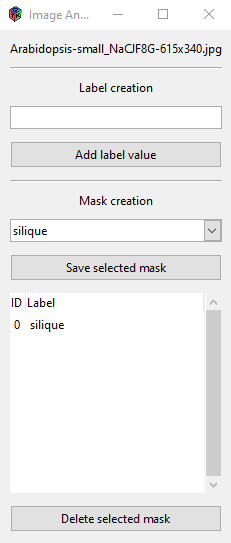

Supplement: giae123_Supplemental_Files [file giae123_supplemental_files.zip › supplementary_fig1_gui_v2_windows.png]

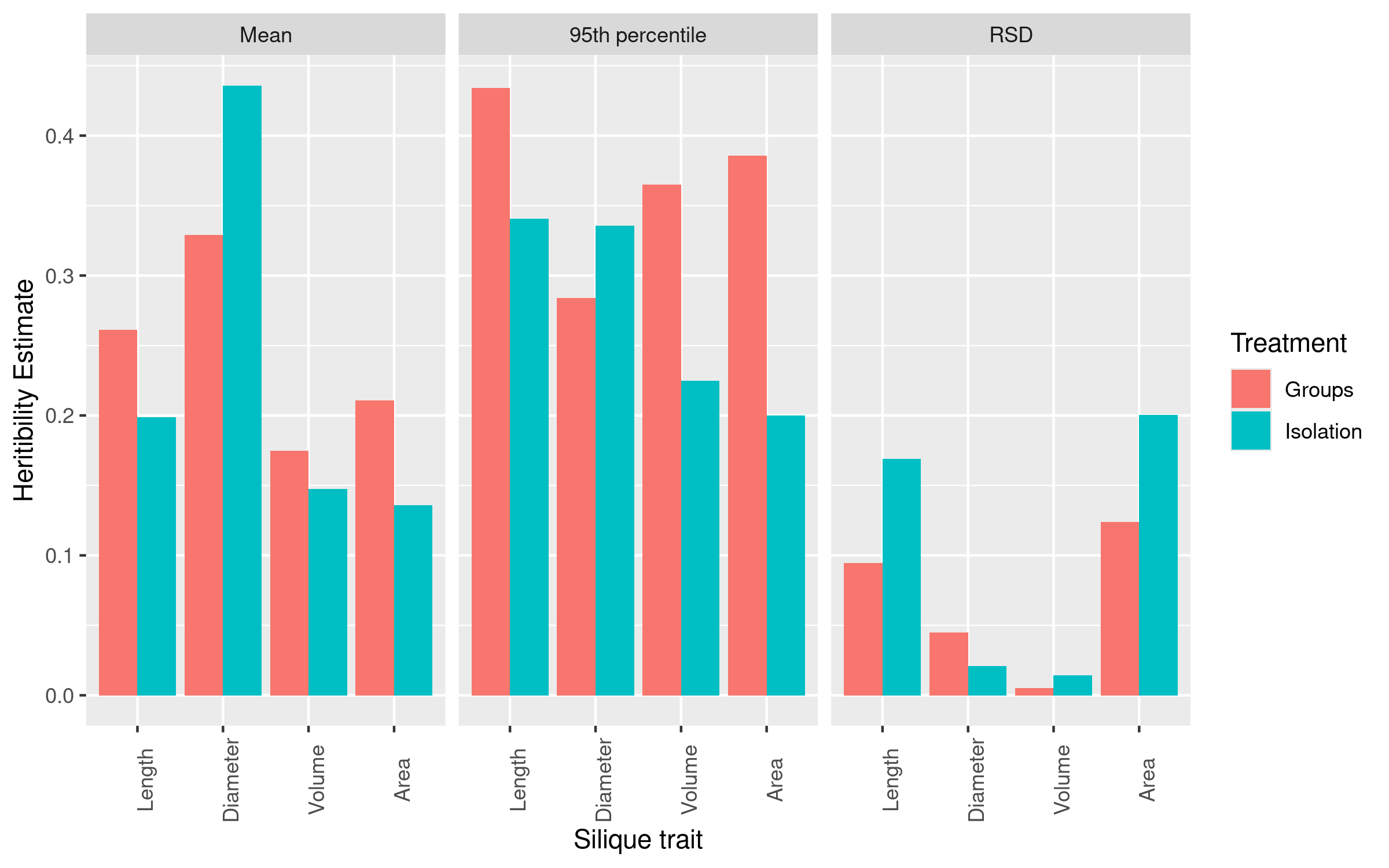

Supplement: giae123_Supplemental_Files [file giae123_supplemental_files.zip › supplementary_fig2_herit.png]

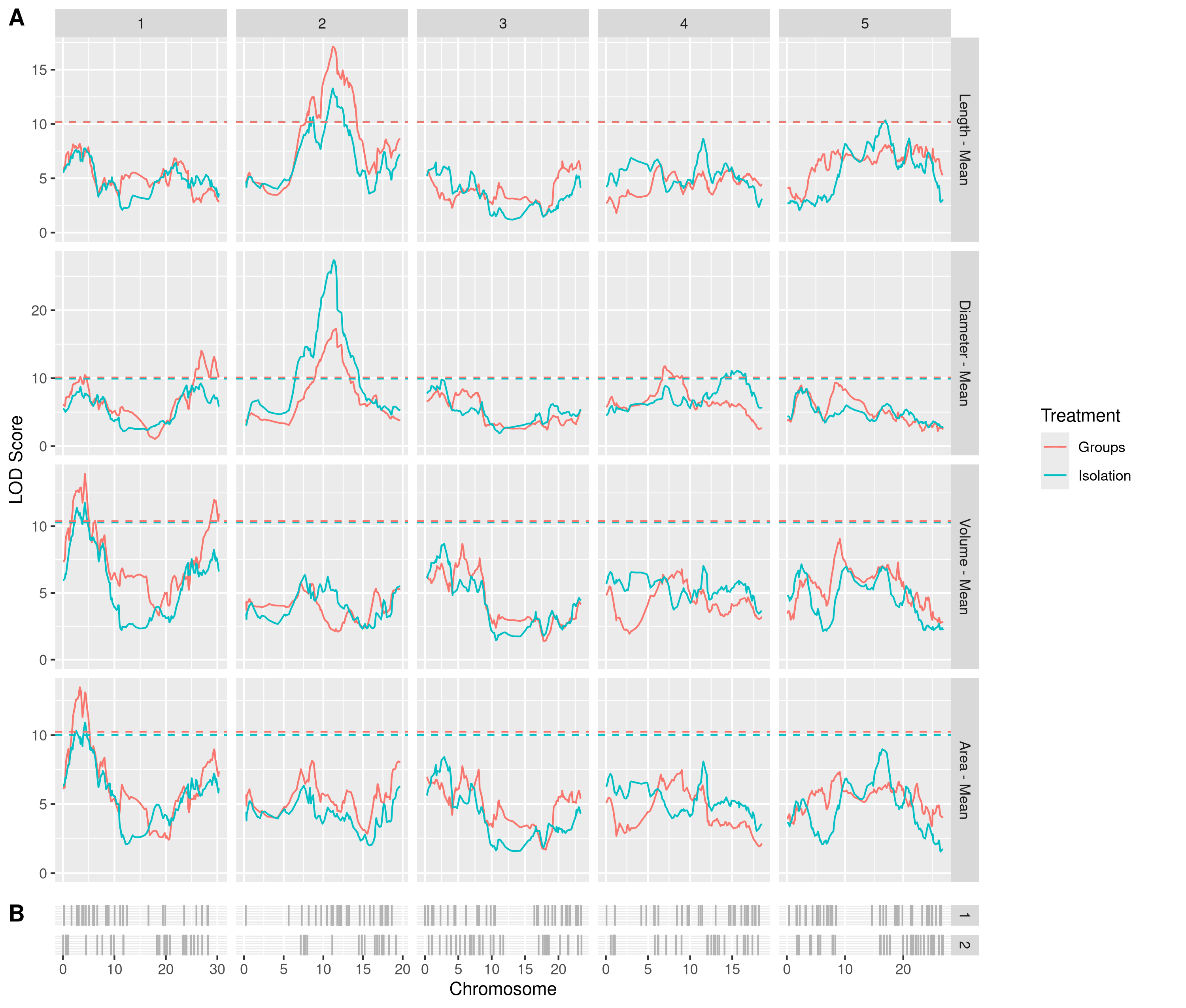

Supplement: giae123_Supplemental_Files [file giae123_supplemental_files.zip › supplementary_fig3_scans_mean.png]

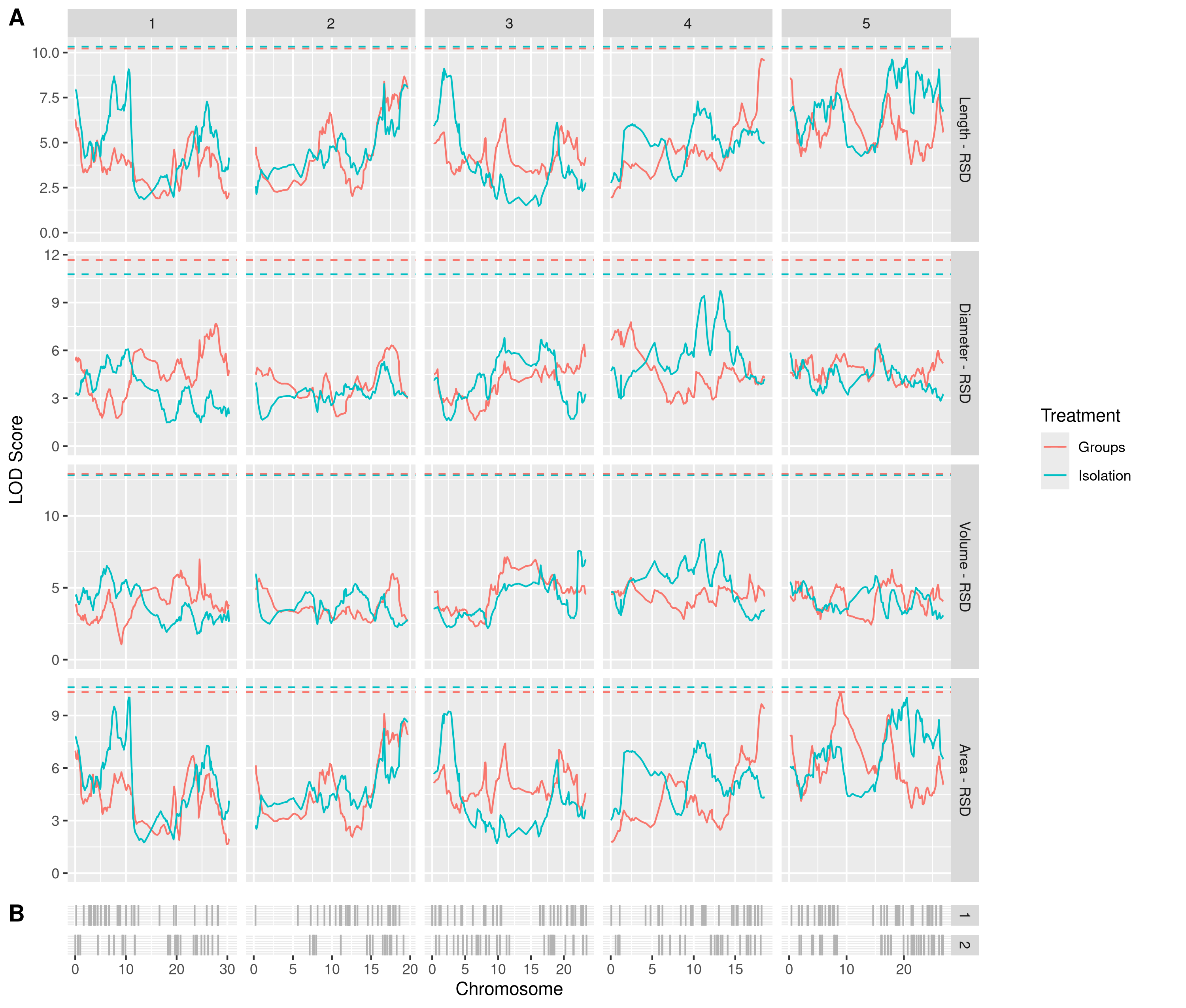

Supplement: giae123_Supplemental_Files [file giae123_supplemental_files.zip › supplementary_fig4_scans_rsd.png]
