# Supplementary material for: Unlocking the power of AI for phenotyping fruit morphology in Arabidopsis
Source: Gigascience. 2025 Feb 12;14:giae123. doi: 10.1093/gigascience/giae123 (PMC11816797; doi:10.1093/gigascience/giae123)
Supplement: giae123_GIGA-D-24-00464_Revision_1 [file giae123_giga-d-24-00464_revision_1.pdf]

GigaScience

Unlocking the Power of AI for Phenotyping Fruit Morphology in Arabidopsis

--Manuscript Draft--

**Manuscript Number:**	GIGA-D-24-00464R1
**Full Title:**	Unlocking the Power of AI for Phenotyping Fruit Morphology in Arabidopsis
**Article Type:**	Research
**Funding Information:**	Biotechnology and Biological Sciences Research Council (FoodBioSystems Doctoral Training Partnership (DTP)) — Mr Kieran Atkins<br>Aberystwyth University (AberDoc) — Dr Gina Garzón-Martínez<br>Medical Research Council (MR/T043253/1) — Dr Andrew Lloyd<br>European Regional Development Fund (ERDF) via the Welsh Government (Supercomputing Wales project) — Not applicable<br>Engineering and Physical Sciences Research Council (EP/Y005430/1) — Prof John Hub Doonan<br>Horizon 2020 Framework Programme (EPPN2020 (No 731013)) — Prof John Hub Doonan<br>Biotechnology and Biological Sciences Research Council (National Capability in Plant Phenotyping (BBS/E/W/0012844A)) — Prof John Hub Doonan
**Abstract:**	Deep learning can revolutionise high-throughput image-based phenotyping by automating the measurement of complex traits, a task that is often labour-intensive, time-consuming, and prone to human error. However, its precision and adaptability in accurately phenotyping organ-level traits, such as fruit morphology, remain to be fully evaluated. Establishing the links between phenotypic and genotypic variation is essential for uncovering the genetic basis of traits and can also provide an orthologous test of pipeline effectiveness. In this study, we assess the efficacy of deep learning for measuring variation in fruit morphology in Arabidopsis using images from a multiparent advanced generation inter-cross (MAGIC) mapping family. We trained an instance segmentation model and developed a pipeline to phenotype Arabidopsis fruit morphology, based on the models outputs. Our model achieved strong performance with an average precision (AP) of 88.0% for detection and 55.9% for segmentation. Quantitative trait locus (QTL) analysis of the derived phenotypic metrics of the MAGIC population identified significant loci associated with fruit morphology. This analysis, based on automated phenotyping of 332194 individual fruits, underscores the capability of deep learning as a robust tool for phenotyping large populations. Our pipeline for quantifying plant morphological traits is scalable and provides high-quality phenotype data, facilitating genetic analysis and gene discovery, and advancing crop breeding research.
**Corresponding Author:**	Chuan Lu<br>Aberystwyth University<br>Aberystwyth, Ceredigion UNITED KINGDOM
**Corresponding Author Secondary Information:**	
**Corresponding Author's Institution:**	Aberystwyth University
**Corresponding Author's Secondary Institution:**	
**First Author:**	Kieran Atkins
**First Author Secondary Information:**	
**Order of Authors:**	Kieran Atkins

Powered by Editorial Manager® and ProduXion Manager® from Aries Systems Corporation

	Gina Garzón-Martínez
	Andrew Lloyd
	John Hub Doonan
	Chuan Lu
**Order of Authors Secondary Information:**	
**Response to Reviewers:**	See attached response letter.
**Additional Information:**	
**Question**	**Response**
Are you submitting this manuscript to a special series or article collection?	No
**Experimental design and statistics**<br><br>Full details of the experimental design and statistical methods used should be given in the Methods section, as detailed in our Minimum Standards Reporting Checklist. Information essential to interpreting the data presented should be made available in the figure legends.<br><br>Have you included all the information requested in your manuscript?	Yes
**Resources**<br><br>A description of all resources used, including antibodies, cell lines, animals and software tools, with enough information to allow them to be uniquely identified, should be included in the Methods section. Authors are strongly encouraged to cite Research Resource Identifiers (RRIDs) for antibodies, model organisms and tools, where possible.<br><br>Have you included the information requested as detailed in our Minimum Standards Reporting Checklist?	Yes
**Availability of data and materials**<br><br>All datasets and code on which the conclusions of the paper rely must be	Yes

Powered by Editorial Manager® and ProduXion Manager® from Aries Systems Corporation

either included in your submission or deposited in publicly available repositories (where available and ethically appropriate), referencing such data using a unique identifier in the references and in the "Availability of Data and Materials" section of your manuscript.<br><br>Have you have met the above requirement as detailed in our Minimum Standards Reporting Checklist?	
GigaScience has policies and guidelines in place for the use of generative AI-writing tools such as ChatGPT. If you have used such writing tools to assist with writing the manuscript this must be declared and cited in the text. Authors should not list AI-writing tools and other AI-assisted technologies as an author or co-author and should acknowledge that they are fully responsible for text generated or refined by AI-writing tools.<p><br><br>A summary of use (particularly in the introduction or among methods) needs to be included at the end of the paper, and the outputs should also be included as a supplementary file hosted in GigaDB or other open repositories. Please read our guidelines for more information. <p><br><br>By submitting to GigaScience, you are aware of the journal's AI-writing tools policy, and if you have declared use of such tools below, you have acknowledged this where appropriate in your manuscript and have made a summary of use and outputs available. <p><br><b>AI-assisted writing tools have been used in the preparation of this manuscript?	No

Powered by Editorial Manager® and ProduXion Manager® from Aries Systems Corporation

Manuscript

Click here to access/download;Manuscript;AraFruitMorphQTL_r1.pdf

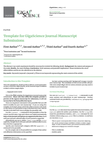

(GIGA)[n] SCIENCE

GigaScience, 2024, 1–16

doi: xx.xxxx/xxxx
Manuscript in Preparation
Paper

PAPER

Unlocking the Power of AI for Phenotyping Fruit Morphology in Arabidopsis

Kieran Atkins[1], Gina A. Garzón-Martínez[3], Andrew Lloyd[1], John H. Doonan[1,*] and Chuan Lu[2,*]

[1]National Plant Phenomics Centre, IBERS, Aberystwyth University, Aberystwyth SY23 3EE, UK and [2]Computer Science Department, Aberystwyth University, Aberystwyth SY23 3EA, UK and [3]Centro de Investigación Tibaitatá, Corporación Colombiana de Investigación Agropecuaria (Agrosavia), Mosquera, Cundinamarca, Colombia

[*]Address correspondence to: jhd2@aber.ac.uk, cul@aber.ac.uk

Abstract

Deep learning can revolutionise high-throughput image-based phenotyping by automating the measurement of complex traits, a task that is often labour-intensive, time-consuming, and prone to human error. However, its precision and adaptability in accurately phenotyping organ-level traits, such as fruit morphology, remain to be fully evaluated. Establishing the links between phenotypic and genotypic variation is essential for uncovering the genetic basis of traits and can also provide an orthologous test of pipeline effectiveness. In this study, we assess the efficacy of deep learning for measuring variation in fruit morphology in Arabidopsis using images from a multiparent advanced generation inter-cross (MAGIC) mapping family. We trained an instance segmentation model and developed a pipeline to phenotype Arabidopsis fruit morphology, based on the models outputs. Our model achieved strong performance with an average precision (AP) of 88.0% for detection and 55.9% for segmentation. Quantitative trait locus (QTL) analysis of the derived phenotypic metrics of the MAGIC population identified significant loci associated with fruit morphology. This analysis, based on automated phenotyping of 332194 individual fruits, underscores the capability of deep learning as a robust tool for phenotyping large populations. Our pipeline for quantifying pod morphological traits is scalable and provides high-quality phenotype data, facilitating genetic analysis and gene discovery, and advancing crop breeding research.

Key words: Plant phenotyping; Arabidopsis; Fruit morphology; Instance segmentation; Deep learning, QTL analysis, MAGIC population

Introduction

Phenotyping fruit morphology

Complex traits, such as organ size, are closely related to plant productivity in both crop breeding and natural settings. These traits influence ecological processes like plant-plant competition and reproductive success. Seed production is an important component of reproductive success, intimately associated with specialised organs called fruits that develop from a specialised organ within the flower called the gynoecium. Fruits are the main harvested product from row crops such as wheat, rice, and oats as well as from fruit crops such as strawberries, tomatoes, bush fruits, and apples. Quantity, quality and other fruit-related traits are therefore of immense agronomic importance, underpinning the economics of modern agriculture. Climate change, increased pest diversity and an ever-growing population threaten reliable food production, therefore it is becoming increasingly crucial to identify and phenotype these traits at scale, with a view to integrating knowledge gained into crop production [1].

The factors that influence fruit size and shape are many and can interact in a complex manner (reviewed by Cucinotta et al. [2]). Intrinsic factors include plant signalling molecules such as auxin and gibberellin whose levels depend on genetic, environmental and developmental variables. The potential ultimate fruit size is partly set before pollination by, for example, the number of ovules present

Compiled on: December 14, 2024.
Draft manuscript prepared by the author.

in the gynoecium, but the realisation of fruit size often depends on the success of pollination. For example, the dry simple fruits found in the Brassica or cabbage family usually will develop only if pollination has been successful [3]. Within a given species, fruit size and particularly fruit length typically reflects the number of seeds present [4, 5]. The Brassica family also has sophisticated genetic resources available, both in crops such as rapeseed/canola [6] and in undomesticated research models such as Arabidopsis [7]. These resources and associated mapping techniques provide an orthologous means of testing the biological significance of any novel phenotyping system.

Arabidopsis thaliana produces many fruits, termed siliques, whose small size and fragile structure make it difficult to collect morphological phenotype data by hand consistently and reliably. Unlike many domesticated crops that retain their seeds, Arabidopsis is a wild plant where the seed pods shatter soon after ripening. With often more than 100 siliques per plant and experimental designs delivering large populations (thousands to tens of thousands) of plants for genetic and ecological studies, new phenotyping approaches are essential to capture this data in the short time window available. RGB imaging provides a low-cost solution that minimises handling of the individual fruits, provided the computer vision tools are capable of extracting phenotypic information, again at low cost and in a timely manner.

Image-based plant phenotyping offers a non-destructive, high-throughput approach to data collection. While 3D imaging, hyperspectral imaging, and multimodal techniques provide rich information, 2D RGB imaging remains a practical choice due to its simplicity and efficiency [8, 9]. However, accurate object detection and measurement within plant images can be challenging due to issues such as organ overlap and the projection of 3D structures into 2D.

Classical computer vision (CV) uses hand-crafted algorithms to analyse images. Classical CV methods can successfully measure fruit morphology [10, 11] and have been used to generate phenotypes for genetic analysis [12, 13]. However, classical CV is typically only suitable for collecting traits from simple scenes (i.e isolated plants and organs; highly colour-differentiated organs) and is usually unable to robustly handle complex scenes, such as overlapping samples or the presence of other plant-biomass. Large-scale phenotyping, especially for fragile or hard-to-harvest fruits, is more effective while the fruits remain attached to the stem. Deep learning offers an alternative approach to classical CV when analysing complex scenes. Deep learning models are trained to replicate the relationship between inputted images and hand-collected training data, to then infer outputs on novel, unseen samples. This also allows deep learning to be trained to handle detection and segmentation with specimens still in their biological contexts. Deep learning models are inherently scalable and can be trained or re-trained to cope with subjects beyond their initial training and have been used successfully in high-throughput phenotyping [14, 15].

Deep learning-based approaches

Convolutional neural networks (CNN) are widely used in image-based deep learning. CNNs consist of layers of trainable image filters that progressively learn to extract features for analysis. Lower-level features, such as edges and corners, are captured in earlier layers, while higher-level features, such as object parts and semantic concepts, are developed in the later layers. These higher-level features are then used for higher-level tasks, such as image classification. For example, Atila et al. [16] developed an EfficientNet CNN to classify the presence of different diseases on the leaves of multiple species, achieving a final accuracy of 99.91% in their most successful model. Similarly, Morshed et al. [17] used DenseNet to assess the quality of harvested fruit, classifying each image as having either good, bad or mixed-quality fruit across 6 species, with an accuracy of 99.67%.

However, extracting quantitative data on individual organs that are still attached to the plant requires a different model architecture. For example, Hamidinekoo et al. [18] created DeepPod, a CNN pipeline for counting Arabidopsis siliques in images of flattened fruit-bearing branches. Using DenseNet, overlapping grid patches within an image were classified as either background, base, body or tip of a silique, and the individual siliques were reconstructed by joining the segmented pixels in a post-processing step. While valuable phenotype data can be extracted from organ detection and localisation, it cannot be used to capture detailed morphometric data on the detected objects. To more effectively phenotype the morphology of the fruit, instance segmentation models may offer a better solution.

Instance segmentation combines both object detection with semantic segmentation, detecting objects and segmenting each instance of an object from the background. Instance segmentation models are also designed to be agnostic to the specific shapes of objects being detected. Two predominant methods employed in instance segmentation models are single-stage and two-stage. Single-stage methods, like YOLACT, an extension of YOLO, predict object bounding boxes and segmentation masks in a single pass through the network [19, 20]. Variants of these architectures have been applied in plant phenotyping for rapid inference, such as identifying tomato fruit and cherry branches for robotic fruit picking and real-time detection and counting of objects like tea buds [21]. However, these methods often prioritize real-time application over achieving the highest accuracy.

Two-stage methods, in contrast, have an initial object proposal stage followed by a classification stage, this tends to result in more precise detections [22], whilst still generating outputs in negligible time. Segmentation is then performed within predicted bounding boxes or cluster pixel projections into instances. Mask R-CNN [23], one of the most prominent two-stage architectures, has been used for phenotyping tasks such as detecting leaf disease [24] and barley seed segmentation from dense scenes [25]. Two-stage methods can be improved by integrating attention mechanisms, like transformer architectures [26, 27], to enable spatially aware feature learning and potentially more accurate segmentation by eliminating the region proposal phase. Although transformers have been used in 3D segmentation of point clouds, such as extraction of rapeseed pod traits [28], their higher computational demands and greater need for training data can make them less practical than R-CNN models in certain situations [27]. Alternatively, multi-stage methods like Cascade Mask R-CNN can enhance accuracy by progressively refining detection [29, 27], allowing the model to better handle variations in scale and occlusion commonly found in agricultural imagery. In this study, we choose Cascade Mask R-CNN for instance segmentation.

Deep learning has been successfully applied in phenotyping and the results effectively linked to the genome. For example, Wang et al. [30] used a CNN to identify the developmental stages of wheat in the field, evaluating morphology without precise measurement and subsequently used these results for QTL analysis, detecting novel epistatic interactions affecting flowering time. SegFormer [31], a transformer-based semantic segmentation model, was used to segment rice panicles from shoots. The segmented images enabled extraction of plant-level traits that were then used to identify QTLs associated with variation in yield. However, the model only segmented panicle from background within the images limiting its resolution for extraction individual organ traits such as grain length and width, which were instead obtained through manual phenotyping [32]. Thus, it remains uncertain whether deep learning can generate high-quality phenotype data on complex morphological traits, such as fruit size and shape, that can be reliably associated with genetic variation and environmental conditions. For a truly high-throughput phenotyping system, the data should be collected with minimal human intervention and have sufficient resolution to

detect small differences between genotypes as well as treatment-induced variation.

To formally assess whether deep learning can be used for precise morphological quantification, we developed an image-based fruit phenotyping pipeline leveraging an instance segmentation deep learning model to extract morphological trait variability and associate this with the genome. We utilised images of fruit-bearing stems of *Arabidopsis thaliana*, harvested from a multiparent advanced generation inter-cross (MAGIC) population [33] where samples had been grown as either single plants in isolation or groups of four under competitive conditions. This dataset enabled us to assess the ability of a deep learning phenotyping system to accurately capture organ-level morphometric traits in an experimental setting and at a scale useful for the identification of causative genetic variation. To reduce manual effort, we developed a data annotation tool based on the image manipulation software GIMP, speeding up the data annotation process. We trained a Cascade Mask R-CNN model to detect and accurately segment Arabidopsis siliques. The phenotypic data derived from these segmentations were used for QTL analysis, showing that deep learning-derived phenotype data can be confidently associated with known QTL. Furthermore, potential novel genomic regions affecting trait variation were identified, demonstrating the potential of deep learning for gene discovery.

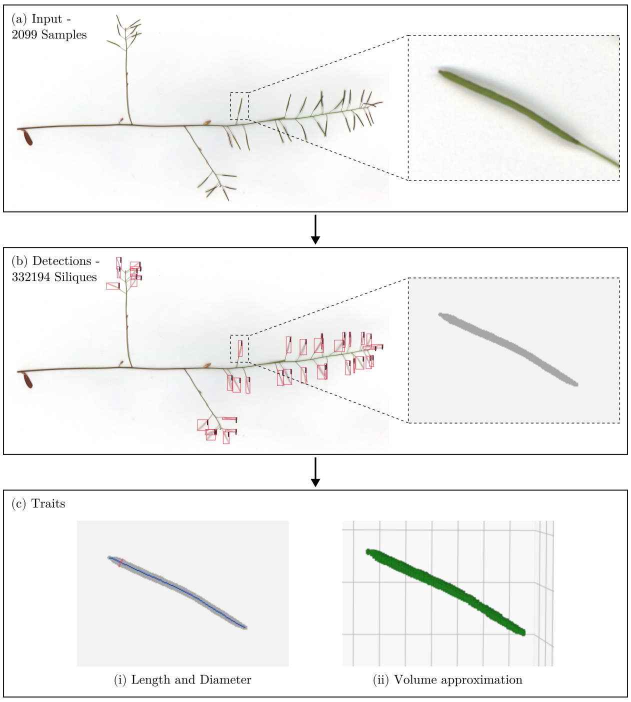

Figure 1. Workflow - Panel (a) shows an example input image alongside a zoomed cutout showing an example silique. Panel (b) shows the output from the network on example input, alongside an example generated mask. Panel (c) shows derived traits from the generated mask, left shows the length (blue, spinal line) and the maximum diameter (red, lateral line) overlaid on the mask, right shows a 3D visualisation of the silique, based on the diameter values along the length of the silique. 2099 samples representing 362 lines are processed by the pipeline, outputting 332194 independently measured siliques.

Materials and Methods

Image data

In this study, we utilised images of stem material from an experiment investigating the genetic basis of sibling-sibling competition in *Arabidopsis thaliana*, using 485 lines from the MAGIC population by Biernaskie & Garzón-Martínez et al. [34]. The population was a multiparent advanced generation inter-cross (MAGIC) consisting of a core set of 527 recombinant inbred lines (RILs), derived from 19 intermated accessions Kover et al. [33]. Plants were grown in pots containing either single plants (isolation) or in groups of four of the same line (groups). A total of 2801 pots represented 485 genotypes across two treatments and three experimental batches, with three replicates per batch, were initially collected. Of these, 2099 were ultimately used for analysis (details in the subsection on phenotypic data extraction).

Mature inflorescences and stems from a single pot, containing either one plant or four plants depending on the treatment, constituted a single sample for imaging. Thus all the stems from a single pot were flattened and scanned at 300 dpi using a flatbed scanner. 7132 images were taken and saved as PNG files. The full experimental design behind experiment is described by Biernaskie & Garzón-Martínez et al. [34]. For our study, we trained a model on a subset of these images, performed inference on the full dataset and created a phenotyping pipeline to extract morphometric traits from the outputs. This workflow is shown in Figure 1.

Image annotation using the GIMP image annotator

Effective training of instance segmentation networks often requires that objects are well annotated within each image. Typically, image annotation involves drawing a polygon along the boundaries of each object. However, our image collection tends to feature clear boundaries between the plant biomass and the background, which can be exploited using classical computer vision algorithms.

We evaluated various existing segmentation annotation tools. Many popular tools, such as VGG Image Annotator (VIA) [35], rely on manual polygon annotation without offering any computer vision assistance. While tools like CVAT [36] provide integration with deep learning models like segment anything model (SAM [37]) for automatic segmentation based on keypoints or bounding boxes, as well as some basic CV functionalities via OpenCV, we found that these approaches yielded poor segmentation results with imprecise borders for our specific dataset. Therefore, we explored the tools available in the GNU Image Manipulation Program (GIMP) and found that its suite of selection tools could quickly create high-quality segmentations. To further streamline this process, we developed a custom plug-in named the GIMP Image Annotator (GIÀ), which allows users to save selected regions as mask images, along with metadata such as object classes, in a designated folder.

GIÀ features an intuitive Graphical User Interface (GUI) that integrates seamlessly with GIMP, making it a robust training data collection tool. This interactive tool applies existing computer vision algorithms that rely on colour similarity to segment specific structures, such as siliques, within images (known as Fuzzy Select within the software package). GIÀ facilitates the annotation of well-separated, unoccluded siliques. For partially occluded siliques, typically only the ambiguous, occluded region needs hand-drawn boundaries to accurately define the object. This tool significantly reduces the effort required for precise annotation of each object within an image. Using GIÀ, we produced a dataset of 55 images with 2940 annotated siliques.

We employed an amodal instance segmentation approach, which involves segmenting both visible and occluded pixels of an object [38]. By saving masks separately for each instance, the approach allows overlapping regions to be attributed to multiple instances. This approach is particularly useful for scenarios where objects overlap and partially occlude each other, providing a more comprehensive representation of the scene.

Model development

Dataset train and test split

To evaluate the model's outputs on unseen samples, we randomly split the annotated dataset into an 80% training set and a 20% testing set, resulting in 44 training images with 2282 individual silique annotations and 11 testing images with 652 silique annotations.

Cascade Mask R-CNN model training

To capture morphometric data, we used an instance segmentation deep learning model, Cascade Mask R-CNN [29, 23], an improvement over Mask R-CNN by utilising a cascading architecture. This architecture introduces a series of cascaded region of interest (RoI) heads, each trained on progressively harder examples, by using larger IoU thresholds for bounding box detection, leading to improved performance, especially for difficult objects.

The model is capable of both detecting each object (silique) and outputting a mask that denotes the pixels belonging to that specific object instance. These outputted masks form the basis for our phenotype data. Underlying Cascade Mask R-CNN, we used a RegNetX 1.6GF backbone [39] with a feature pyramid network. We initialised the network with weights pre-trained on COCO [40]. COCO is a commonly used, high-quality dataset, and models trained on COCO have already learnt strong lower-level object detection features that form a strong model initialisation point. We initialise the weights without any parameter freezing to allow the model to adapt to the largely different domain of our dataset.

The model was then trained on the training set for 36 epochs, using stochastic gradient descent with a learning rate of 0.01. We found that these parameters allowed the model to converge well on our dataset without overfitting. We included an initial linear learning rate warm-up for the first 100 iterations, starting at 0.00001, and ending at the learning rate of 0.01. This was chosen to stop the sharp initial updates from the initial COCO weights, which can lead to instability [41]. The learning rate was set to decay by a factor of 0.1 at epochs 24 and 33 to allow for fine-tuning later in the training. Stochastic gradient descent also included a momentum value of 0.9 and a weight decay value of 0.0001. The model was trained for 36 epochs with 2x NVIDIA A100 48 GB GPUs and batch size 1, resulting 792 training iterations, taking 1 hour and 21 minutes.

The images within our dataset were too large to fit on the GPU at native resolution during training, so to reduce memory usage whilst preserving silique detail, images were scaled to 4590×3240, 90% of the original size. As the size of the siliques within these images already very small, we found downsampling the size of the images further resulted in poor segmentation results in the higher precision $AP_{0.75}$ metric. We inferred that the higher resolution was therefore necessary to achieve the quality of segmentation and fine detail required in the ultimate phenotype data for relation to the genome. Moreover, while alternative solutions, such as dividing images into smaller patches, could preserve resolution and detail for siliques, they introduce complexity in splitting and reassembling, particularly for future studies focusing on positional branch-level traits. Thus, we chose a higher resolution (compared to the popular setting of less than 1000×1000) that balances image quality with the capabilities of our available GPU resources.

Model evaluation for instance segmentation

We evaluated the model's success in two independent ways: first, by comparing its outputs on previously unseen samples with manually annotated ground truth data; second by evaluating the model's phenotypic data using QTL analysis to identify phenotype-genotype correlations.

Evaluating the model on the unseen test data requires direct comparison of the model's predicted outputs against the hand-collected annotation data. Predicted objects (represented by a bounding box or mask) are considered correctly identified if the intersection-over-union (IoU) between the prediction and the ground truth exceeds a certain threshold. Model detection is categorised as either true positives (TP) or false positives (FP) depending on whether they align with ground-truth annotations for a given IoU threshold. Any annotations the model failed to detect are considered false negatives (FN). We calculate precision (proportion of correctly identified objects TP against all detected objects $TP + FP$) and recall (proportion of correctly identified objects TP to the total number of objects within the sample $TP + FN$) across all detected siliques for both object detection and instance segmentation. We report Average Precision (AP) and Average Recall (AR), representing the average precision or recall values across IoU thresholds of 0.5, 0.75 and the range from 0.5 to 0.95 at 0.05 increments. These metrics are standard in evaluating object detection models and allowed us to check if the model detected all objects within the image, without littering the image with spurious detections.

Phenotypic data extraction

Silique morphological traits

The outputted masks from the instance segmentation model are used to calculate several metrics representing silique morphology. The calculated traits are outlined in Table 1 and visualised in Figure 1. Silique length represents the spinal line, between the pedicel-silique junction to the tip of the silique (remains of the stigma), while the silique diameter represents the maximum width. The projected area (referred to hereafter as "area") is the area of the mask outputted by the model.

The silique volume is based on the Euclidean distance transform values computed from the silique mask. For each point along the central length line (spinal line) of the silique, the distance transform is calculated as the distance to the nearest border of the mask along a line orthogonal to the length line. These values serve as the radius, r, of a series of 1-voxel high cylinders, with volume $\pi r^2 h$, where $h = 1$, centred on each point of the spinal line. Summing the volumes of all cylinders along the spinal line approximates the total silique volume, assuming a cylinder-like morphology where each cross-section is circular with a radius equal to the distance transform.

Phenotypic metrics at the sample level

Prior to QTL analysis, we employed a two-level filtering process to ensure that the extracted phenotypic data were biologically relevant. The first level was applied to the pots (i.e., samples), excluding aberrant samples unrelated to the quality of the model predictions. The second level was applied to the siliques, removing model-detected siliques based on the quality of the generated masks to improve the accuracy of the extracted traits.

The pot level filtering was necessary because, in the original experiment that produced the image data, some individual plants exhibited unusually large numbers of aborted fruits. The cause was unclear, as no specific genotype or growing location followed this pattern. Therefore, adhering to the original experimental protocol [34], any individual plant with more than two branches showing a high number of aborting fruits had all replicate samples of that same genotype removed. 702 samples were therefore removed from the QTL analysis, resulting in a dataset of 2099 samples representing two treatments of 362 genetically distinct MAGIC lines.

Next, we programmatically examined the structure of each silique mask and filtered out masks with multiple connected components, ensuring each mask represented a single connected shape. For length calculations, we verified that the generated skeletons were continuous lines without significant spurs (i.e. exceeding 5 pixels in length). This ensured that the masks conformed to the expected cylindrical morphology and filtered out aberrant masks, for example, masks covering more than a single detected silique.

We extracted silique morphology features from the filtered silique masks, including length, diameter, volume, and area (see Figure 1). For each sample, we calculated various aggregate silique

Trait	Abbr.	Description
Silique area	SA	The raw binary mask output from the network, calculated as the number of segmented pixels, converted into mm^2. Illustrated as grey region in Figure 1 (b)
Silique length	SL	Skeletonisation (using Zhang and Suen [42] thinning algorithm) of the binary mask to define a median line representing the primary structure. This algorithm erodes edges of a binary mask, until only a single, spinal line of pixels remains. The Euclidean distance of the line of pixels approximates the length of the silique, converted in *mm*. Illustrated as a blue spinal line of the pod in Figure 1 (c) (i)
Silique diameter	SD	Calculation of the Euclidean distance transform of the object, finding the largest value along the skeletonised length line, and taking double that value to be an approximation of diameter (or the largest distance to the nearest edge among all points along the length), converted into *mm*. Illustrated as a red lateral line in Figure 1 (c) (i)
Silique volume	SV	Approximation of the silique volume, based on calculating the volume of a series of 1-voxel high cylinder/disk at each point along the spinal line, with the sum approximating the full silique volume, converted into mm^3. 3D illustration in Figure 1 (c) (ii)

Table 1. Individual silique traits - Names, abbreviations and descriptions of each of the traits derived from a single silique.

morphological trait statistics, including mean, percentiles, and relative standard deviation (*RSD*, the ratio of standard deviation to the mean). *RSD* provides a measure of the overall spread of each silique morphology trait within a sample, a potentially desirable trait for breeding. We explored different percentiles (from 95th to 5th) of fruit morphology traits, representing the best and worst fruits produced.

QTL analysis

To assess the impact of competition on silique morphology traits at the population level, we conducted a paired-sample t-test on sample-level data, disregarding genotypic variation. This analysis allowed us to evaluate the utility of the phenotype data before examining genetic differences.

For the QTL analysis, we utilised the sample-level silique metrics (including mean, percentiles, and *RSD*) across all samples of each treatment and genotype. We calculated heritability estimates of the aggregate metrics and found the P_{95} phenotypes had the highest heritability estimate in all the silique traits for both treatments. The 95th percentile phenotype also represents the best a plant or group of plants can produce which is a phenotype of interest in breeding applications. We therefore focused primarily on this phenotype for the QTL analysis. QTL mappings were performed using linear mixed models, with the kinship matrix calculated using the leave-one-chromosome-out (LOCO) method, unless otherwise specified. We estimated the parental effects of each of the founder alleles at putative QTL by fitting a single-QTL model at the QTL position and outputting the estimated regression coefficients of each of the founder alleles. The genetic map used was constructed by Kover et al. [33], consisting of 1250 markers across the 5 chromosomes. 274, 210, 248, 228 and 290 markers for each chromosome respectively, with an average spacing between markers of 0.4 cM, and a maximum gap of 4.5 cM. To account for potential batch effects in the original experiment we included batch as a covariate in the analysis. The Bayes credible interval with a 0.95 probability was calculated to estimate the probable region containing each QTL. Confidence thresholds were calculated using permutation tests where number of permutations = 1000 and $p < 0.05$.

To identify genes related to silique development, we utilised the GOSLIM ontology system [43] to collect gene ontology terms related to fruit development, organ development and organ morphogenesis (GO:001015, GO:009402, GO:1905392 - Set 1) and seed development (GO:0048316 - Set 2), including their child terms. With these ontological terms, we searched the TAIR9 database [7] for genes with known effects on fruit phenotypes. The specific ontologies used are listed in Supplementary Table 3. Genes within the Bayes credible interval were cross-referenced with our detected QTL, and the gene nearest to the QTL peak was identified.

Metric	$AP_{0.5:0.95}$	$AP_{0.5}$	$AP_{0.75}$	$AR_{0.5:0.95}$
Detection	88.0	95.8	94.5	90.7
Segmentation	55.9	94.2	66.0	61.5

Table 2. Detection and Segmentation *AP* and *AR* results - IoU threshold values denoted in subscript.

Software

Models were created and trained using Python library `MMDetection` v3.3 [44], built upon `PyTorch` v2.2.1 [45]. Model phenotyping pipeline was built upon Python libraries `scikit-image` v0.22.0 [46] and `OpenCV` v4.9.0 [47]. QTL analysis was performed using `R/qtl2` v0.36 [48], genomic data was provided by `R/atMAGIC` v0.1.0 [49], which in turn was retrieved from R. Mott's and P. Kover's server (`http://mtweb.cs.ucl.ac.uk/mus/www/magic/`).

Results

Model performance on silique detection and segmentation

We initially evaluate the direct outputs of the model, which consist of predicted bounding boxes and associated masks for the detected siliques for given thresholds, reporting precision and recall metrics for both silique detection and segmentation, as outlined in Section .

Table 2 shows the detection and segmentation *AP* and *AR* results from the test split of the annotated dataset. For detection, the model achieves an average precision of over 95% for siliques when an IoU threshold of 0.75 is used on the detected boxes. We observe similar performance for the average of IoU thresholds ranging from 0.50 to 0.95, achieving a precision of 88% and recall of 91%. For segmentation, the model achieves an average precision of 66% for siliques when an IoU threshold of 0.75 is used on the segmentation mask. The model also achieves a segmentation IoU score of over 0.50 in 94% of detected siliques. The model successfully detects and creates quality segmentations of the majority of siliques within the unseen test set. We show example qualitative results in Figure 2, where we see consistent, accurate segmentation of each silique, even in situations of partial occlusion.

We also note the amodal instance segmentation performance of our model. We found that in situations of occlusion, our model was typically capable of segmenting both siliques amodally, even more so if the siliques were perpendicular to each other. We believe this is due to the visual clues of tips and bases of the siliques informing the detector, and the fact that perpendicular occlusion fairs better is due to the intersection area of the two objects being reduced, giving more information to the model to consider them as two distinct instances. As long as neighbouring bounding boxes don't largely overlap, so as not to get suppressed by non-max suppression, and there are enough visual clues in the image, the model is capable of handling amodal segmentation in situations of occlusion.

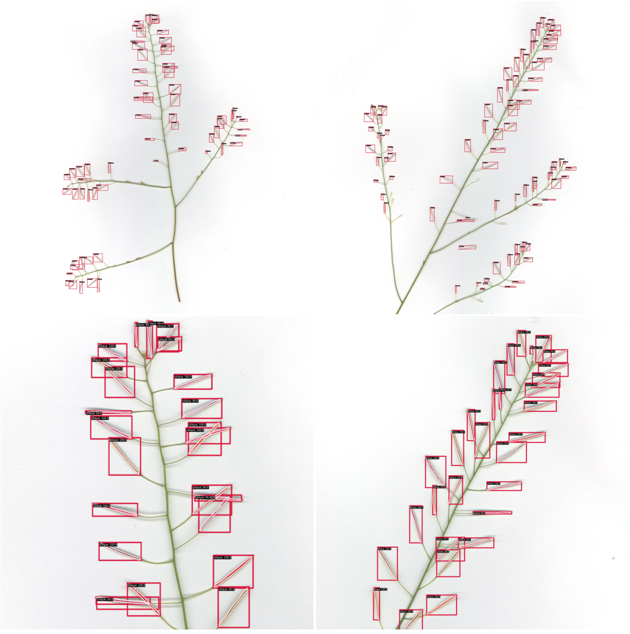

Figure 2. Example qualitative results on unseen images from full *Arabidopsis thaliana* dataset. Red boxes represent detections of siliques. The red mask within each box detection represents the detected mask for that detected silique. Percent values tied to each detection represent the confidence score. Upper row shows full stem material, lower row shows zoomed regions.

Treatment effects on the distributions of phenotype data

Given the model's strong performance in detection and segmentation, we then evaluated the plant-level phenotype data extracted from the model predictions to assess its ability to detect treatment effects, specifically the impact of competition on silique morphology within the MAGIC population.

Figure 3 shows the distribution of the 95th percentile phenotypes of the four silique traits across the population of 362 lines for each treatment. For the length, volume and area phenotypes, we see a normal-like distribution for both treatments, with a reduction in the overall values for plants grown in competition. This indicates that for the population, the group growth treatment reduces silique length and volume. Diameter has much less spread than the other phenotypes but this is, at least partially, the result of the imaging process. The diameter values are extremely small (approximately 5-10 pixels across) so any minor variation will be binned into the available pixels, and therefore metrics such as P_{95} that take a value at a specific location in the distribution will result in selecting the same value for many samples, resulting in a metric with less resolution. However, the P_{95} metric still best represents the maximum potential of each plant, therefore, we chose to move forward using it for our analysis.

We performed a paired-sample *t*-test using the sample-level summary statistics, specifically the 95th percentile of silique morphology traits, for each line and treatment. For the phenotypic metrics (95th percentile, mean, and *RSD*) of length, diameter and volume, we found a statistically significant difference between the two treatments at $p < 0.05$ across the population, except for the 95th percentile of diameter. The *p* values for each phenotypic metric are provided in Supplementary Table 2. This indicates a statistically significant difference in most of these phenotypic metrics between the two treatment distributions at the population level.

QTL mapping, parental effects and alignment of identified QTL to known genes

While the response of the entire population to competition was clear and significant, there was extensive phenotypic variation among the genotypes for all traits. We therefore used QTL analysis to test whether the deep learning-derived phenotypes were suitable for genetic analysis.

Figure 4 shows the QTL mapping of length, diameter, volume and area P_{95} phenotypes under both treatments, with the calculated significance thresholds represented as a horizontal dashed line. Genes with relevant ontologies related to fruit development, organ development, and organ morphogenesis are shown in grey (complete list is available in supplementary data). A complete list of detected QTL for the 95th percentile phenotypes is provided in Table 3 alongside 95% Bayes credible intervals.

For the silique length phenotype, strong QTL signals were detected on chromosome 2 with significant peaks, ranging from 11.143 to 11.588 Mb for both treatments, suggesting a treatment-invariant QTL (designated as SL2-1). The closest gene with relevant ontology to the QTL peak is *ER*, or *erecta*. The *erecta* mutation, at 11.208 to 11.215 Mb, produces short fat club-like siliques [50] - a decreasing effect on length and an increasing effect on diameter. To determine the parental source of the variation at this locus for silique length and silique width, we fitted a single-QTL model using a linear mixed model and calculated the parental effect of each of the founder alleles. Figure 5A shows the effect of the founder alleles on the length P_{95} phenotype. The LER (Landsberg Erecta) founder allele provides the main decreasing effect for SL2-1 with a smaller contribution from Can-0 about the QTL peak. The *ER* gene (8.4kbs away from the peak) is shown as a dashed black line. Figure 5B illustrates the opposite, with an increasing effect for diameter (SD2-1) for the same founder allele.

Additionally, there are two significant peaks on chromosome 1 for both the volume and area P_{95} phenotypes within the region of 3.101 – 3.398 Mb and peaks at 4.249 Mb. These loci show a significant association for the group treatment only, suggesting the P_{95} phenotypes of these traits are treatment-sensitive. The first locus related to volume, SV1-1 with a peak at 4.249 Mb, had the closest gene *ESR1*. The second locus, SV1-2 with a peak at at 29.389 Mb and a Bayes credible interval of 28.579 – 29.814 Mb, had no genes in our ontology list near it, suggesting a novel locus. It is worth noting,

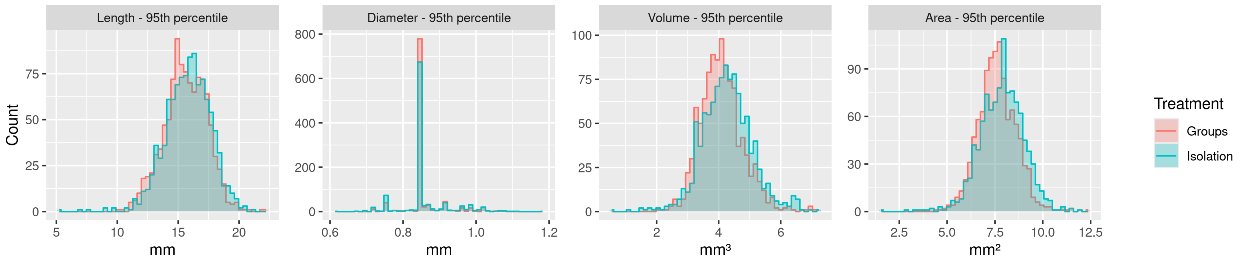

Figure 3. 95th percentile (P_{95}) phenotype distributions - Histograms of the distribution of the P_{95} for length, diameter, volume and area, with each treatment overlaid. Group treatment is represented in red and isolation treatment is represented in blue. Data from the filtered population of the 362 lines. Note: All figures have free axis scales

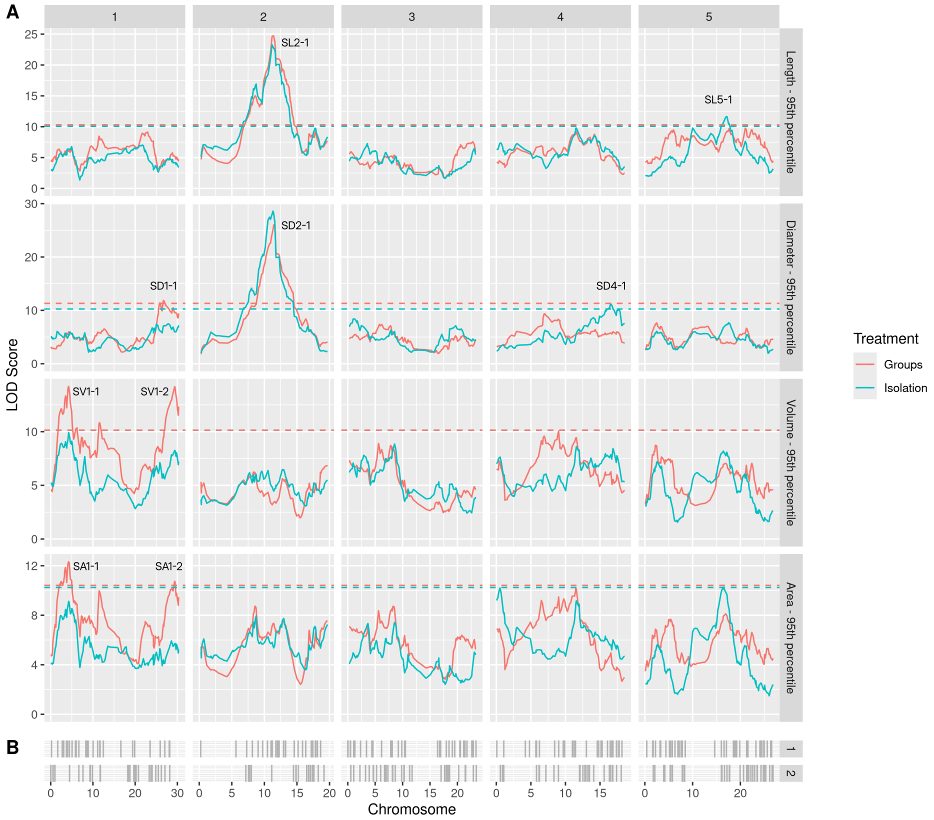

Figure 4. A. QTL mapping for 95th percentile (P_{95}) phenotypes for length, diameter, volume and area. Horizontal dashed lines represent the significance threshold calculated using permutation tests (number of permutations = 1000, $p < 0.05$). Detected QTL designations are written and positioned next to associated peaks. Group treatment is represented in red and isolation in blue. B. Genes with relevant ontologies related to fruit development, organ development and organ morphogenesis (Set 1) & seed development (Set 2) shown as vertical lines indicating the starting position of the gene. Set number is shown in facet title on right side. Data are from the filtered population of 362 lines.

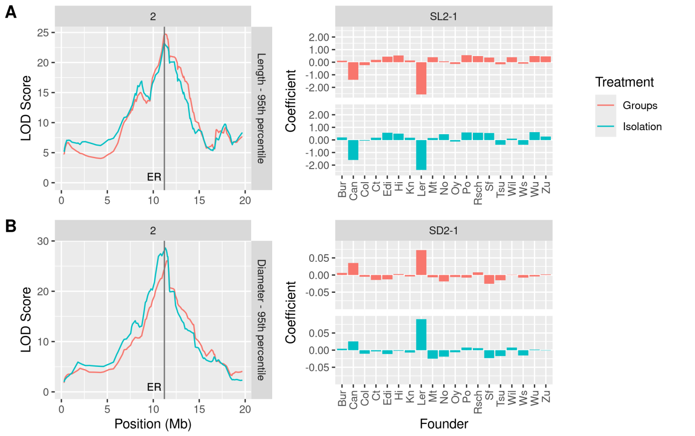

Figure 5. QTL mapping outputs and parental effect coefficients for each of the founder alleles for (A) Length - P_{95} and (B) Diameter - P_{95} phenotypes comparing group and isolation treatments. QTL analysis outputs (left) display the peak positions of QTL on chromosome 2, while the corresponding QTL names are shown above the parental effect coefficient bar plots (right). The position of the gene *ER* is indicated by a solid vertical line. Data are from the filtered population of 362 lines.

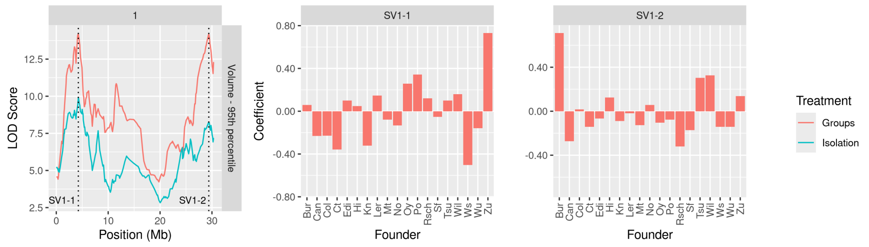

Figure 6. QTL mapping outputs and parental effect coefficients for the significant QTL associated with the Volume - P_{95} phenotype, comparing group and isolation treatments. The peak positions of detected QTL on chromosome 1 are indicated by vertical dashed lines. Parental effect coefficient bar plots for the two significant QTL in the group treatment are provided. No significant QTLs were found for the isolation treatment at these positions. Data are from the filtered population of 362 lines.

Trait	QTL	Treatment	Chromosome	LOD	Interval (Mb)	Peak (Mb)	Nearest Gene
Silique length	SL2-1	Isolation	2	23.25	11.143 - 11.588	11.200	ER
	SL2-1	Groups	2	24.73	11.143 - 11.541	11.324	ER
	SL5-1	Isolation	5	11.65	16.186 - 17.676	17.169	SCP2
Silique diameter	SD2-1	Isolation	2	28.60	10.793 - 11.480	11.324	ER
	SD2-1	Groups	2	26.06	11.324 - 11.588	11.541	PGY1
	SD1-1	Groups	1	11.85	25.735 - 28.976	26.682	SERK1
	SD4-1	Isolation	4	11.20	15.787 - 17.804	16.575	ADC2
Silique volume	SV1-1	Groups	1	14.22	03.398 - 04.569	04.249	ESR1
	SV1-2	Groups	1	14.19	28.405 - 29.814	29.389	×
Silique area	SA1-1	Groups	1	12.33	02.548 - 04.927	04.249	ESR1
	SA1-2	Groups	1	10.73	27.852 - 29.893	29.389	BZR1

Table 3. List of all significant 95th percentile (P_{95}) phenotype QTL for all traits. Bayes credible interval calculated with probability = 0.95. Nearest gene is calculated by the closest gene to peak within the Bayes credible interval. Data from the filtered population of the 362 lines.

however, that the highly related area phenotype also detected a QTL at the same peak as SV1-2 but with a wider credible interval, and found the nearby gene *BZR1* from our ontology list. Figure 6 shows the significant QTL associated with the volume P_{95} phenotype.

Discussion

This report describes the successful application of an instance segmentation model based on Cascade Mask R-CNN, along with a pipeline for quantitative phenotyping of *Arabidopsis thaliana* siliques at scale. Our deep learning approach enabled the extraction of morphological traits which, when combined with genetic data, led to the identification of high-confidence QTL. As far as we know, this is the first time that fruit morphological data, generated by a deep learning system, has been successfully linked to genomic variation. Many previous studies have identified QTL influencing fruit morphology, in a wide variety of crops [12, 13, 51, 52, 53, 54]. However, these tend to involve manual or simple classical CV-based measurements of isolated fruits. For example, Gegas et al. [51] used a high-throughput imaging approach with harvested grain (botanically, a single seeded fruit) to dissect the genetic architecture of kernel variation in breadwheat and its close relatives.

Where fruits are easily harvested and handled, for example, tomatoes, squashes and bananas amongst others, it is possible to phenotype manually and at a sufficient scale to undertake genetic analyses. This is even possible with minor crops such as Chinese bayberry, *Myrica rubra* [55]. However, many types of fruit, particularly from undomesticated species such as the model experimental species Arabidopsis, are inherently difficult to harvest due to the inherent fragility of the ripe fruits. Therefore, whole stems carrying the siliques were harvested to minimise shattering, the samples were then pressed before imaging to maximise the accuracy of trait measurements. Alternative methods, such as multi-view RGB imaging of whole plants followed by structure-from-motion 3-D modelling [56, 57] were considered too slow to enable data capture from the entire population. Likewise, CT scanning of plants with long thin stems that needed careful immobilisation was not feasible at scale.

While it is technically possible to extract morphometric data manually from physical samples, it is difficult to complete this sort of operation at scale and within a timeframe where such samples typically retain their structural coherence. Thus, a high resolution image dataset of experimental samples was created and stored within days of harvest, thereby immortalising the information for later extraction and analysis. An alternative to imaging would be to employ a relatively large number of trained staff but this is often not financially feasible even for crops. One person, however, can achieve imaging of large sample numbers at minimal cost as it requires ubiquitous low-cost hardware. Phenotype data such as fruit position, angle and colour exist within the images used within this study, but would need pipelines developed for high-throughput extraction. This potential of further data extraction is only possible due to the imaging process, and tools that computer vision provides. As computer vision tools progress, more phenotype data will be able to be extracted from these populations without the cost of setting up new experiments.

The simplicity and speed of 2D imaging and flat-bed scanning allowed us to critically evaluate the use of deep learning phenotyping on a large population that was designed to address fundamental biological questions [34]. However, due to the inherent complexity of the material being analysed, and the relatively small training dataset, the deep learning model generated unwanted artefacts. To address this, we employed basic filtering techniques. We exclude all masks that return more than one connected component which typically occurs when a bounding box has enclosed more than one silique or parts of 2 or more neighbouring siliques. In such cases, the mask generation branch of the network attempts to include those pixels in the detection. We also excluded masks that have more than one skeletal line (after pruning smaller skeletal branches), as this, like multiple connected components, only typically occurs when regions of neighbouring siliques have been included in the

mask output. Overall, these artefacts occur in 7.3% of detections on (median) average per image. Appropriate filtering ensured that the final dataset was suitable for downstream genetic analysis.

Collecting high resolution silique morphological data at this scale is not typically possible using manual measurements. Typical manual QTL approaches tend to estimate genetic potential from a relatively small subset of hand-collected fruits [5, 58, 59, 60]. Our approach allowed us to analyze the entire silique output from each sample, generating various statistical measures, including mean, percentiles, and relative standard deviation (*RSD*). Notably, the 95th percentile of each silique trait gave the strongest overall heritability (ranging from 0.27-0.43). This was confirmed by QTL mapping of the mean versus P_{95} metrics (Figure 4 and Supplementary Figure 3): while the overall landscape was similar, the P_{95} phenotype gave higher confidence scores. As our system generates morphological data on very large numbers of individually detected fruit with defined criteria for including or excluding defined components of data, it removes subjective bias from the analysis allowing full exploration of different facets of fruit quality.

Uniformity of fruit size within a cultivar is another desirable trait depending on the crop. Thus, millers prefer uniform grain size in many small-grain cereals for processing reasons [61] whereas "standard" scales for uniformity are defined in breeding many fruits. Notably in tomato, uniformity has been reported as highest in small-fruited varieties [62], but particular alleles of quantitative trait locus *qMULTIPLE INFLORESCENCE BRANCH 2* (*qMIB2*), a *SPATULA*-like gene, can contribute to fruit size stability in larger fruited strains [63]. To assess uniformity, we used *RSD* but this had low heritability and did not reveal any significant QTL, despite the presence of a significant treatment effect that affected the QTL mapping of *RSD*, as reflected in LOD (logarithm of the odds) scores in certain genomic regions (Supplementary Figure 4). Variation in silique morphology is perhaps influenced more by development than genetics or treatment as the inflorescence stem is determinate, with later flowers producing shorter siliques [64].

Conventional wisdom suggests that small training datasets, like ours, typically lead to overfitting in deep learning models. However, despite being trained on only 44 images and around 2000 annotated siliques, our model generalised well to the remaining dataset. This is likely due to the uniform background and the high number of single-class objects per image. The abundance of objects per image also makes the loss value at each training step more informative, which is crucial given the variability in siliques' shape, size, openness, and fertility within the same plant. Our image and data acquisition strategy enables effective fine-tuning of a COCO pre-trained model for our specific task. We chose to use a smaller backbone for the model, ultimately deciding to use RegNetX 1.6GF, a model with approximately half the parameter count of ResNet-50. The RegNet family of CNNs are designed to have more efficient sized depth and width to reduce redundancy in parameters. We found that using a smaller backbone resulted in equal or improved detection and segmentation results compared to it's larger parameter-count siblings. This implies the larger models overfit easily on this constrained dataset and the regularisation effect of reducing the model size benefits this task. Reducing model size also allowed more memory to fit the large images during training.

Conclusion

This paper has presented a deep learning pipeline capable of phenotyping morphometric traits of siliques of *Arabidopsis thaliana*. It demonstrates an innovative application of deep learning models as a phenotyping tool, focusing on the capability of relating phenotypic variation to the genome. In particular, our work expands the knowledge of what is possible with automatically collected phenotype data through deep learning, revealing that high-confidence QTL can be identified on the basis of morphology phenotypes from a deep learning model. The systematic verification results demonstrate that our deep learning phenotyping data have the quality and accuracy required for genetic analysis of continuous morphometric characteristics of attached plant organs, in the context of high-precision quantitative phenotyping.

The phenotypic and genotypic datasets, as well as the code and models developed in this study, are fully available at the open data repository. We ensure reproducibility by adhering to FAIR principles and providing step-by-step documentation, allowing for ease of replication by the scientific community. In keeping with the principles of open science, our pipeline not only provides immediate insights into silique morphology but also acts as a foundational tool for future open-source development. Researchers working in different phenomics environments can modify our pipeline, allowing for extended applications beyond Arabidopsis thaliana. The scalability of our pipeline underscores its potential for application in other large-scale phenotyping studies, such as those in crop breeding programs or ecological field studies. As deep learning continues to evolve, models like ours are well-positioned to be further reinforced to handle the increasing data complexity and volume inherent to high-throughput plant phenotyping.

Our phenotyping approach has direct implications for precision agriculture and breeding, allowing for the high-resolution quantification of key agronomic traits. Adaptation to other plant species with similar architectures, especially Brassica seed crops, should be relatively straightforward. While our model generalizes well on this relatively focused dataset, future research could explore more diverse datasets or augment existing data to improve robustness. Additionally, these image collections contain a wealth of additional interesting information so expanding the pipeline to detect other plant structures (such as petioles, stems and flowers) and features (e.g., branch angles, numbers and organ positions) could enhance its applicability to broader plant phenomics studies. We hope this extensive image dataset and associated genetic tools will motivate others to re-use this data.

Availability of source code

MorphPod: Deep learning phenotyping of Arabidopsis fruit morphology

- https://github.com/kieranatkins/silique-detector/
- Operating system: Platform independent
- Programming language: Python, R
- Other requirements: see public environment file
- released under GNU GPL v3
- bio.tools: biotools:morphpod
- RRID: MorphPod (RRID:SCR_026174)

GIMP Image Annotator (GIÀ): a lightweight GIMP plug-in for computer vision-assisted image annotation

- https://github.com/kieranatkins/gimp-image-annotator
- Operating system: Platform independent
- Programming language: Python
- Other requirements: GIMP
- released under GNU GPL v3
- bio.tools: biotools:gimp_image_annotator
- RRID: gimp_image_annotator (RRID:SCR_026175)
- Workflow Hub: 10.48546/workflowhub.workflow.1229.1

Data availability

The dataset for model development and validation with QTL analysis are available in an open data repository, DOI:10.20391/283ce324-6a96-4cc8-8168-51f48354f7cf under CC0 licence.

Declarations

List of abbreviations

MAGIC	Multiparent Advanced Generation Inter-Cross
QTL	Quantitative Trait Locus
CNN	Convolutional Neural Network
R-CNN	Region Based Convolutional Neural Network
RIL	Recombinant Inbred Line
COCO	Common Objects in COntext
RSD	Relative Standard Deviation (also Coefficient of Variation)
LOD	Logarithm of the Odds
AP	Average Precision
AR	Average Recall
IoU	Intersection-over-Union (also Jaccard Index)

Consent for publication

Not applicable.

Competing Interests

The authors declare that they have no competing interests.

Funding

We acknowledge the support of the EPPN2020 grant (No. 731013), the National Capability in Plant Phenotyping (BBS/E/W/0012844A), the Miscanthus AI project (EPSRC EP/Y005430/1), the Supercomputing Wales project and the AccelerateAI project (part-funded by ERDF through the Welsh Government). KA was supported by a PhD studentship from the UKRI BBSRC FoodBioSystems Doctoral Training Partnership (DTP), grant number BB/T008776/1; GGM was supported by the AberDoc scholarship from Aberystwyth University; AL is funded through a UK Research and Innovation Future Leaders Fellowship (MR/T043253/1).

Acknowledgements

We acknowledge Dr Jay Biernaskie from University of Oxford for allowing us to use the experimental dataset prior to publication. Thanks to Ralph Lidner and Al-Fida M. Ahmed for their assistance with data collection.

Author Contributions

KA annotated the dataset, trained the model, created the phenotyping pipeline and performed the analyses. KA wrote the manuscript. GGM designed and performed the original experiment and collected the image dataset. AL oversaw the QTL and gene ontology analyses. JHD and CL obtained the funding and supervised the project. All authors proofread and agreed on the manuscript.

Supplementary

Metric	Symbol	Description
Mean	μ	Mean average silique length, diameter, volume and area for each sample, mean averaged for all reps
95th percentile	P_{95}	The 95th percentile of silique length, diameter, volume and area values for each sample, mean averaged for all reps
Relative standard deviation	*RSD*	The standardised measure of the distribution of a population ($RSD = \frac{\sigma}{\mu}$), also known as the coefficient of variation, calculated for silique length, diameter, volume and area for each sample, mean averaged for all reps.

Supplementary Table 1. Phenotypes - Descriptions of calculated phenotypes for each genotype

Phenotype	Metric	p
Length	P_{95}	1.098×10^{-6}
Diameter	P_{95}	0.211
Volume	P_{95}	4.531×10^{-11}
Area	P_{95}	5.443×10^{-13}
Length	μ	7.979×10^{-6}
Diameter	μ	7.484×10^{-5}
Volume	μ	2.356×10^{-6}
Area	μ	2.264×10^{-16}
Length	*RSD*	6×10^{-3}
Diameter	*RSD*	3.222×10^{-11}
Volume	*RSD*	4.443×10^{-7}
Area	*RSD*	3.14×10^{-2}

Supplementary Table 2. Paired-sample t-test results for the population-level analysis of the effect of competition for the P_{95} phenotypes, comparing the distributions of both treatments. Data from the filtered population of the 362 lines.

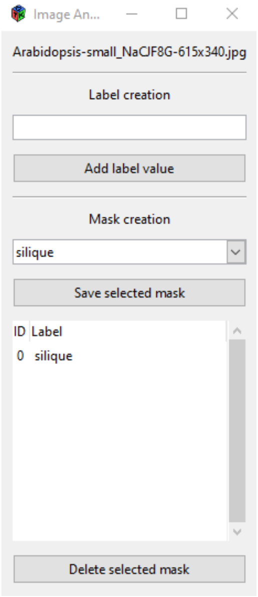

Supplementary Figure 1. Data Collection GUI - A screenshot of the GUI running alongside GIMP to manage the collection of training data

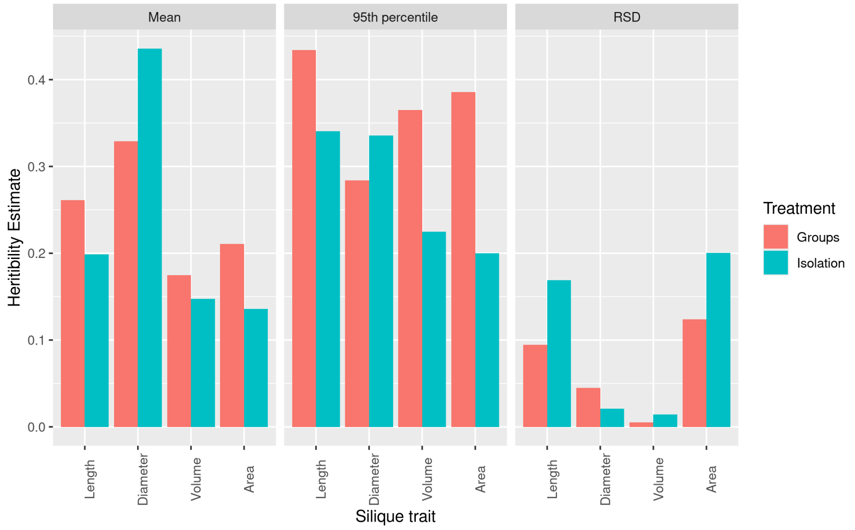

Supplementary Figure 2. Heritability estimations - Estimations of heritability of each of the explored phenotypes, for each silique trait. Calculated via a linear mixed model using an overall kinship matrix, with batch introduced as a covariate. Each silique trait is shown with each phenotype metric derived from the data, including the mean (μ), P_{95} and *RSD*. Data are from the filtered population of the 362 lines.

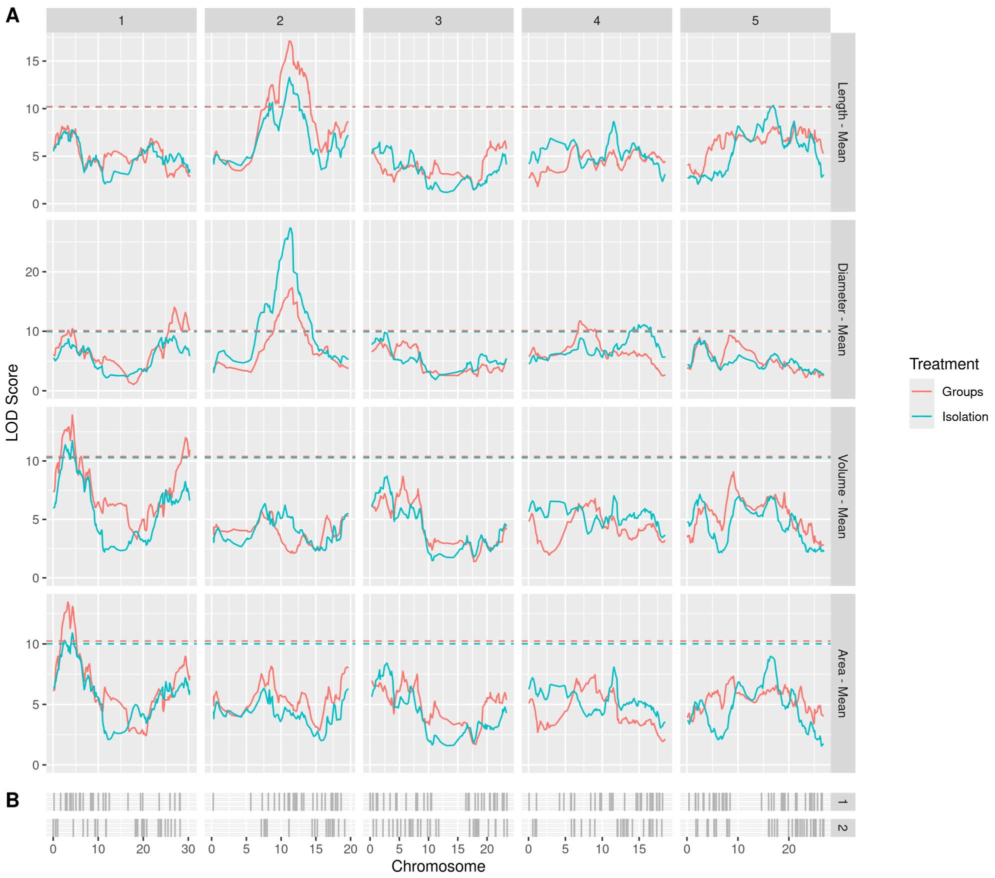

Supplementary Figure 3. A. QTL mapping for mean phenotypes for length, diameter, volume and area. Horizontal dashed lines represent the significance threshold calculated using permutation tests (number of permutations = 1000, $p < 0.05$). B. Genes with relevant ontologies related to fruit development, organ development and organ morphogenesis (Set 1) & seed development (Set 2) are shown as vertical lines indicating the starting positions of the gene. Group treatment is represented in red, and isolation in blue. Data are from the filtered population of the 362 lines.

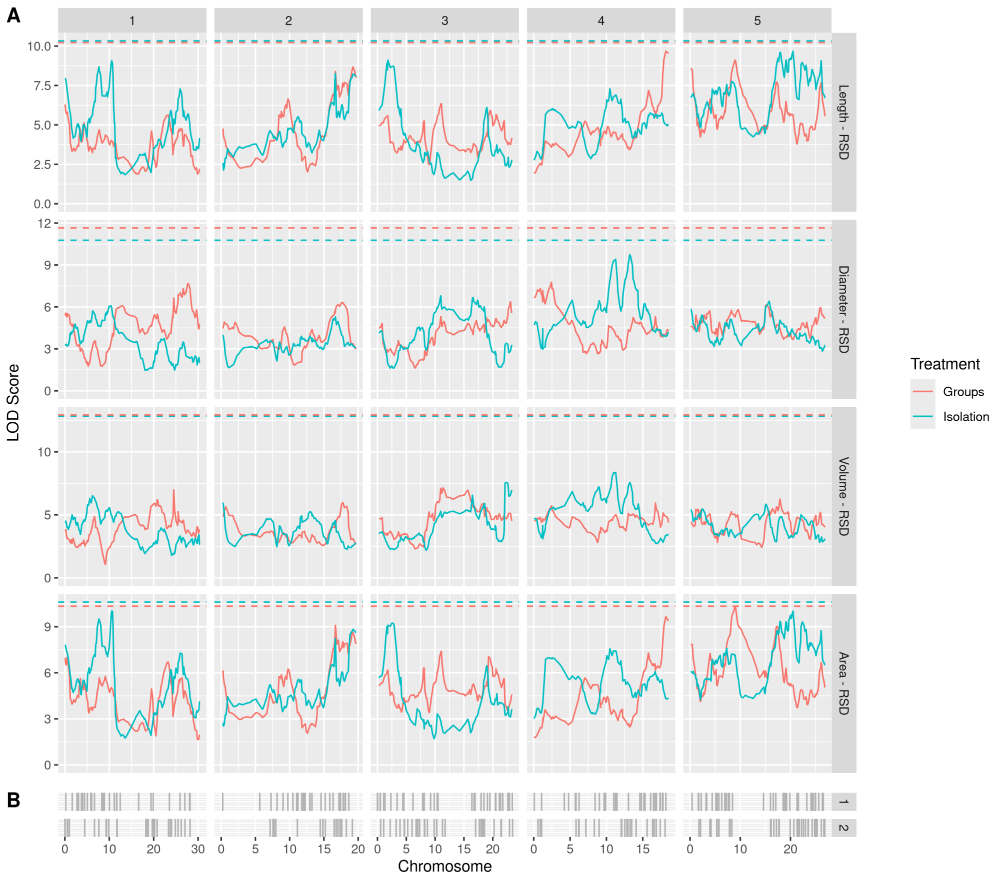

Supplementary Figure 4. A. QTL mapping for relative standard deviation (*RSD*) phenotypes for length, diameter, volume, and area. Horizontal dashed lines represent the significance threshold calculated using permutation tests (number of permutations = 1000, $p < 0.05$). B. Genes with relevant ontologies related to fruit development, organ development and organ morphogenesis (Set 1) & seed development (Set 2) are shown as vertical lines indicating the starting position of the gene. Group treatment is represented in red, and isolation in blue. Data are from the filtered population of the 362 lines.

Set	GO ID	Name
1	GO:0010154	fruit development
1	GO:0048530	fruit morphogenesis
1	GO:0080127	fruit septum development
1	GO:1990058	fruit replum development
1	GO:1990059	fruit valve development
1	GO:0099402	plant organ development
1	GO:0048481	plant ovule development
1	GO:0090696	post-embryonic plant organ development
1	GO:1905392	plant organ morphogenesis
1	GO:0048482	plant ovule morphogenesis
1	GO:0090697	post-embryonic plant organ morphogenesis
1	GO:1905421	regulation of plant organ morphogenesis
1	GO:1905422	negative regulation of plant organ morphogenesis
1	GO:1905423	positive regulation of plant organ morphogenesis
2	GO:0048316	seed development
2	GO:0009960	endosperm development
2	GO:0010431	seed maturation
2	GO:0048317	seed morphogenesis
2	GO:0080050	regulation of seed development
2	GO:0080112	seed growth

Supplementary Table 3. Gene ontologies used to subset list of genes.

References

1. Marsh JI, Hu H, Gill M, Batley J, Edwards D. Crop breeding for a changing climate: integrating phenomics and genomics with bioinformatics. Theoretical and Applied Genetics 2021 Jun;134(6):1677–1690. https://doi.org/10.1007/s00122-021-03820-3.
2. Cucinotta M, Di Marzo M, Guazzotti A, de Folter S, Kater MM, Colombo L. Gynoecium size and ovule number are interconnected traits that impact seed yield. Journal of Experimental Botany 2020 02;71(9):2479–2489. https://doi.org/10.1093/jxb/eraa050.
3. Vivian-Smith A, Luo M, Chaudhury A, Koltunow A. Fruit development is actively restricted in the absence of fertilization in Arabidopsis. Development 2001 06;128(12):2321–2331. https://doi.org/10.1242/dev.128.12.2321.
4. Zhang L, Yang G, Liu P, Hong D, Li S, He Q. Genetic and correlation analysis of silique-traits in Brassica napus L. by quantitative trait locus mapping. Theoretical and Applied Genetics 2011 Jan;122(1):21–31. https://doi.org/10.1007/s00122-010-1419-1.
5. Siles L, Hassall KL, Sanchis Gritsch C, Eastmond PJ, Kurup S. Uncovering Trait Associations Resulting in Maximal Seed Yield in Winter and Spring Oilseed Rape. Frontiers in Plant Science 2021;12. https://www.frontiersin.org/journals/plant-science/articles/10.3389/fpls.2021.697576.
6. Ton LB, Neik TX, Batley J. The Use of Genetic and Gene Technologies in Shaping Modern Rapeseed Cultivars (Brassica napus L.). Genes 2020;11(10). https://www.mdpi.com/2073-4425/11/10/1161.
7. Berardini TZ, Reiser L, Li D, Mezheritsky Y, Muller R, Strait E, et al. The arabidopsis information resource: Making and mining the "gold standard" annotated reference plant genome. genesis 2015;53(8):474–485. https://onlinelibrary.wiley.com/doi/abs/10.1002/dvg.22877.
8. Pieruschka R, Schurr U. Plant Phenotyping: Past, Present, and Future. Plant Phenomics 2019;2019:7507131. https://spj.science.org/doi/abs/10.34133/2019/7507131.
9. Yang W, Feng H, Zhang X, Zhang J, Doonan JH, Batchelor WD, et al. Crop Phenomics and High-Throughput Phenotyping: Past Decades, Current Challenges, and Future Perspectives. Molecular Plant 2020;13(2):187–214. https://www.sciencedirect.com/science/article/pii/S1674205220300083.
10. Berry JC, Fahlgren N, Pokorny AA, Bart RS, Veley KM. An automated, high-throughput method for standardizing image color profiles to improve image-based plant phenotyping. PeerJ 2018 Oct;6:e5727. https://peerj.com/articles/5727.
11. He H, Ma X, Guan H. A calculation method of phenotypic traits of soybean pods based on image processing technology. Ecological Informatics 2022;69:101676. https://www.sciencedirect.com/science/article/pii/S1574954122001261.
12. Diaz-Garcia L, Covarrubias-Pazaran G, Schlautman B, Grygleski E, Zalapa J. Image-based phenotyping for identification of QTL determining fruit shape and size in American cranberry (Vaccinium macrocarpon L.). PeerJ 2018 Aug;6:e5461. https://peerj.com/articles/5461.
13. Zingaretti L, Monfort A, Pérez-Enciso M. Automatic Fruit Morphology Phenome and Genetic Analysis: An Application in the Octoploid Strawberry. Plant Phenomics 2021 05;2021:1–14.
14. Pound MP, Atkinson JA, Townsend AJ, Wilson MH, Griffiths M, Jackson AS, et al. Deep machine learning provides state-of-the-art performance in image-based plant phenotyping. GigaScience 2017 08;6(10):gix083. https://doi.org/10.1093/gigascience/gix083.
15. Gill T, Gill S, Chopra Y, Koff J, Saini D, Sandhu K. A Comprehensive Review of High Throughput Phenotyping and Machine Learning for Plant Stress Phenotyping. Plant Phenomics 2022 04;2:3.
16. Atila Ü, Uçar M, Akyol K, Uçar E. Plant leaf disease classification using EfficientNet deep learning model. Ecological Informatics 2021;61:101182. https://www.sciencedirect.com/science/article/pii/S1574954120301321.
17. Morshed MS, Ahmed S, Ahmed T, Islam MU, Ashikur Rahman ABM. Fruit Quality Assessment with Densely Connected Convolutional Neural Network. In: 2022 12th International Conference on Electrical and Computer Engineering (ICECE); 2022. p. 1–4.
18. Hamidinekoo A, Garzón-Martínez GA, Ghahremani M, Corke FMK, Zwiggelaar R, Doonan JH, et al. DeepPod: a convolutional neural network based quantification of fruit number in Arabidopsis. GigaScience 2020 03;9(3):giaa012. https://doi.org/10.1093/gigascience/giaa012.
19. Bolya D, Zhou C, Xiao F, Lee YJ. YOLACT: Real-Time Instance Segmentation. In: 2019 IEEE/CVF International Conference on Computer Vision (ICCV); 2019. p. 9156–9165.
20. Redmon J, Divvala S, Girshick R, Farhadi A. You Only Look Once: Unified, Real-Time Object Detection. In: 2016 IEEE Conference on Computer Vision and Pattern Recognition (CVPR); 2016. p. 779–788.
21. Li Y, Feng Q, Liu C, Xiong Z, Sun Y, Xie F, et al. MTA-YOLACT: Multitask-aware network on fruit bunch identification for cherry tomato robotic harvesting. European Journal of Agronomy 2023;146:126812. https://www.sciencedirect.com/science/article/pii/S1161030123000801.
22. Carranza-García M, Torres-Mateo J, Lara-Benítez P, García-Gutiérrez J. On the Performance of One-Stage and Two-Stage Object Detectors in Autonomous Vehicles Using Camera Data. Remote Sensing 2021;13(1). https://www.mdpi.com/2072-4292/13/1/89.
23. He K, Gkioxari G, Dollar P, Girshick R. Mask R-CNN. In: Proceedings of the IEEE International Conference on Computer Vision (ICCV); 2017. .
24. Afzaal U, Bhattarai B, Pandeya YR, Lee J. An Instance Segmentation Model for Strawberry Diseases Based on Mask R-CNN. Sensors 2021;21(19). https://www.mdpi.com/1424-8220/21/19/6565.
25. Toda Y, Okura F, Ito J, Okada S, Kinoshita T, Tsuji H, et al. Training instance segmentation neural network with synthetic datasets for crop seed phenotyping. Communications Biology 2020 04;3:173.

26. Cheng B, Misra I, Schwing AG, Kirillov A, Girdhar R. Masked-Attention Mask Transformer for Universal Image Segmentation. In: Proceedings of the IEEE/CVF Conference on Computer Vision and Pattern Recognition (CVPR); 2022. p. 1290–1299.
27. Gu W, Bai S, Kong L. A review on 2D instance segmentation based on deep neural networks. Image and Vision Computing 2022;120:104401. https://www.sciencedirect.com/science/article/pii/S0262885622000300.
28. Du R, Ma Z, Xie P, He Y, Cen H. PST: Plant segmentation transformer for 3D point clouds of rapeseed plants at the podding stage. ISPRS Journal of Photogrammetry and Remote Sensing 2023;195:380–392. https://www.sciencedirect.com/science/article/pii/S092427162200315X.
29. Cai Z, Vasconcelos N. Cascade R-CNN: High Quality Object Detection and Instance Segmentation. IEEE Transactions on Pattern Analysis and Machine Intelligence 2021;43(5):1483–1498.
30. Wang X, Xuan H, Evers B, Shrestha S, Pless R, Poland J. High-throughput phenotyping with deep learning gives insight into the genetic architecture of flowering time in wheat. GigaScience 2019 11;8(11):giz120. https://doi.org/10.1093/gigascience/giz120.
31. Xie E, Wang W, Yu Z, Anandkumar A, Álvarez JM, Luo P. SegFormer: Simple and Efficient Design for Semantic Segmentation with Transformers. CoRR 2021;abs/2105.15203. https://arxiv.org/abs/2105.15203.
32. Geng Z, Lu Y, Duan L, Chen H, Wang Z, Zhang J, et al. High-throughput phenotyping and deep learning to analyze dynamic panicle growth and dissect the genetic architecture of yield formation. Crop and Environment 2024;3(1):1–11. https://www.sciencedirect.com/science/article/pii/S2773126X23000655.
33. Kover PX, Valdar W, Trakalo J, Scarcelli N, Ehrenreich IM, Purugganan MD, et al. A Multiparent Advanced Generation Inter-Cross to Fine-Map Quantitative Traits in Arabidopsis thaliana. PLOS Genetics 2009 07;5(7):1–15. https://doi.org/10.1371/journal.pgen.1000551.
34. Biernaskie JM, Garzón-Martínez GA, Corke FMK, Doonan JH. Under revision: Uncovering the hidden genetic basis of plant competitiveness and group productivity. Proceedings of the Royal Society B: Biological Sciences 2024;.
35. Dutta A, Zisserman A. The VIA Annotation Software for Images, Audio and Video. In: Proceedings of the 27th ACM International Conference on Multimedia MM '19, New York, NY, USA: ACM; 2019. https://doi.org/10.1145/3343031.3350535.
36. CVAT ai Corporation, Computer Vision Annotation Tool (CVAT); 2023. https://github.com/cvat-ai/cvat.
37. Kirillov A, Mintun E, Ravi N, Mao H, Rolland C, Gustafson L, et al. Segment Anything. arXiv:230402643 2023;.
38. Li K, Malik J, Amodal Instance Segmentation; 2016. https://arxiv.org/abs/1604.08202.
39. Radosavovic I, Kosaraju RP, Girshick R, He K, Dollar P. Designing Network Design Spaces. In: Proceedings of the IEEE/CVF Conference on Computer Vision and Pattern Recognition (CVPR); 2020. .
40. Lin TY, Maire M, Belongie S, Hays J, Perona P, Ramanan D, et al. Microsoft COCO: Common Objects in Context. In: Fleet D, Pajdla T, Schiele B, Tuytelaars T, editors. Computer Vision – ECCV 2014 Cham: Springer International Publishing; 2014. p. 740–755.
41. Gilmer J, Ghorbani B, Garg A, Kudugunta S, Neyshabur B, Cardoze D, et al. A Loss Curvature Perspective on Training Instability in Deep Learning. CoRR 2021;abs/2110.04369. https://arxiv.org/abs/2110.04369.
42. Zhang TY, Suen CY. A fast parallel algorithm for thinning digital patterns. Communications of the ACM 1984;27(3):236–239.
43. Berardini TZ, Mundodi S, Reiser L, Huala E, Garcia-Hernandez M, Zhang P, et al. Functional Annotation of the Arabidopsis Genome Using Controlled Vocabularies. Plant Physiology 2004 06;135(2):745–755. https://doi.org/10.1104/pp.104.040071.
44. Chen K, Wang J, Pang J, Cao Y, Xiong Y, Li X, et al. MMDetection: Open MMLab Detection Toolbox and Benchmark. CoRR 2019;abs/1906.07155. http://arxiv.org/abs/1906.07155.
45. Paszke A, Gross S, Massa F, Lerer A, Bradbury J, Chanan G, et al. PyTorch: An Imperative Style, High-Performance Deep Learning Library. CoRR 2019;abs/1912.01703. http://arxiv.org/abs/1912.01703.
46. van der Walt S, Schönberger JL, Nunez-Iglesias J, Boulogne F, Warner JD, Yager N, et al. scikit-image: image processing in Python. PeerJ 2014 6;2:e453. https://doi.org/10.7717/peerj.453.
47. Bradski G. The OpenCV Library. Dr Dobb's Journal of Software Tools 2000;.
48. Broman KW, Gatti DM, Simecek P, Furlotte NA, Prins P, Sen Ś, et al. R/qtl2: software for mapping quantitative trait loci with high-dimensional data and multi-parent populations. bioRxiv 2018;https://www.biorxiv.org/content/early/2018/09/12/414748.
49. Tavares H. atMAGIC: Data package with R/qtl2 objects for the Arabidopsis MAGIC lines; 2024, https://github.com/tavareshugo/atMAGIC, r package version 0.1.0, commit 0b8e985975a0d0277cdc0bb4cc7a0dc39393070b.
50. Torii KU, Mitsukawa N, Oosumi T, Matsuura Y, Yokoyama R, Whittier RF, et al. The Arabidopsis ERECTA gene encodes a putative receptor protein kinase with extracellular leucine-rich repeats. The Plant Cell 1996 04;8(4):735–746. https://doi.org/10.1105/tpc.8.4.735.
51. Gegas VC, Nazari A, Griffiths S, Simmonds J, Fish L, Orford S, et al. A Genetic Framework for Grain Size and Shape Variation in Wheat . The Plant Cell 2010 04;22(4):1046–1056. https://doi.org/10.1105/tpc.110.074153.
52. Pan Y, Wang Y, McGregor C, Liu S, Luan F, Gao M, et al. Genetic architecture of fruit size and shape variation in cucurbits: a comparative perspective. Theoretical and applied genetics 2020;133:1–21.
53. Hussain Q, Zhan J, Liang H, Wang X, Liu G, Shi J, et al. Key genes and mechanisms underlying natural variation of silique length in oilseed rape (Brassica napus L.) germplasm. The Crop Journal 2022;10(3):617–626. https://www.sciencedirect.com/science/article/pii/S2214514121001902.
54. Hong Y, Zhang M, Zhu J, Zhang Y, Lv C, Guo B, et al. Genome-wide association studies reveal novel loci for grain size in two-rowed barley (Hordeum vulgare L.). Theoretical and Applied Genetics 2024;137(3):58.
55. Zhang W, Chen K, Zhang B, Sun C, Cai C, Zhou C, et al. Postharvest responses of Chinese bayberry fruit. Postharvest Biology and Technology 2005;37(3):241–251. https://www.sciencedirect.com/science/article/pii/S0925521405001092.
56. Ghahremani M, Williams K, Corke F, Tiddeman B, Liu Y, Wang X, et al. Direct and accurate feature extraction from 3D point clouds of plants using RANSAC. Computers and Electronics in Agriculture 2021;187:106240. https://www.sciencedirect.com/science/article/pii/S016816992100257X.
57. Ghahremani M, Williams K, Corke FMK, Tiddeman B, Liu Y, Doonan JH. Deep Segmentation of Point Clouds of Wheat. Frontiers in Plant Science 2021;12. https://www.frontiersin.org/journals/plant-science/articles/10.3389/fpls.2021.608732.
58. Zhao X, Yu K, Pang C, Wu X, Shi R, Sun C, et al. QTL Analysis of Five Silique-Related Traits in Brassica napus L. Across Multiple Environments. Frontiers in Plant Science 2021 Nov;12. https://www.frontiersin.org/journals/plant-science/articles/10.3389/fpls.2021.766271/full.
59. Jiao Y, Zhang K, Cai G, Yu K, Amoo O, Han S, et al. Fine mapping and candidate gene analysis of a major locus controlling

ovule abortion and seed number per silique in Brassica napus L. Theoretical and Applied Genetics 2021 Aug;134(8):2517–2530. https://doi.org/10.1007/s00122-021-03839-6.

60. Yu P, Gao Z, Hua Z. Contrasting Impacts of Ubiquitin Overexpression on Arabidopsis Growth and Development. Plants 2024;13(11). https://www.mdpi.com/2223-7747/13/11/1485.
61. Esvelt Klos K, Yimer BA, Howarth CJ, McMullen MS, Sorrells ME, Tinker NA, et al. The Genetic Architecture of Milling Quality in Spring Oat Lines of the Collaborative Oat Research Enterprise. Foods 2021;10(10). https://www.mdpi.com/2304-8158/10/10/2479.
62. Macua JI, Lahoz I, Bozal JM, Arméndariz R. Industrial quality of cherry tomato varieties in Navarre. Acta Horticulturae 2007 11;758:181–184.
63. Sun S, Liu Z, Wang X, Song J, Fang S, Kong J, et al. Genetic control of thermomorphogenesis in tomato inflorescences. Nature Communications 2024 Feb;15(1):1472. https://www.nature.com/articles/s41467-024-45722-0.
64. González-Suárez P, Walker CH, Lock T, Bennett T. FLOWERING LOCUS T-mediated thermal signalling regulates age-dependent inflorescence development in Arabidopsis thaliana. Journal of Experimental Botany 2024 03;75(14):4400–4414. https://doi.org/10.1093/jxb/erae094.

Response to editor and reviewer's comments
Click here to access/download;Personal Cover;Response_letter_r1.pdf

Dear Dr. Nicole Nogoy,

We thank you for the chance to improve the manuscript and the reviewers for their comments and time. We have addressed all points of action made by the reviewers and editors. The major changes include:

- Updated description of the silique volume approximation for clarity
- Included zoomed-in regions of Figure 2 for better visibility
- Justification for our choice of using such high-resolution images
- Clarification as to the size of the different dataset subsets
- Including extra justification for the work, comparing computer vision methods to traditional manual phenotyping (both in the Introduction and the Discussion)
- Collating both the GIMP image annotator software and MorphPod model and phenotyping software into the bio.tools and SciCrunch repositories. We also included the workflow used to create our annotations using the GIMP+GIA software in the workflowhub.eu workflow repository.
- General minor corrections

Appended below, we respond to the reviewers' comments on a point-by-point basis. All relatively major changes made are highlighted in the revised manuscript.

Your sincerely,

Kieran Atkins, John Doonan, and Chuan Lu

on behalf of the co-authors.

Point-by-point Responses (in bold) to the Reviewers' Comments (in italic)

Reviewer #1: The authors present a deep learning approach to the detection and segmentation of Arabidopsis fruit, along with a QTL study linking genotype to fruit phenotyping. The paper is well written and clear, and I believe represents a useful and interesting study in this area. I have some minor queries / suggestions:

- If I understand your experimental numbers correctly, you have produced a set of 55 annotated images, which are split 80:20 into training and testing. This would leave a testing set size of ~11 images, is this correct? It would be helpful to include the exact values in the manuscript. Similarly, you train for 36 epochs, why did you choose this value. If an epoch represents one look over the dataset, with a training set of ~44 images across 4 GPUs, you would require only about 400 training iterations to complete the entire training process, is this correct? This is likely fine, but an estimate of the total training time would be helpful.

Yes, that is correct: the annotated dataset was split into 44 images for training (a total of 2282 instance annotations) and 11 images for testing (652 annotations). We have now included this information in the manuscript in the section "Model evaluation for instance segmentation".

Training was stopped at 36 epochs because we observed that the model had converged with the training and validation loss values becoming stable by this point. We acknowledge that this does not seem so many iterations over such a small dataset, and we discuss this some more in our final paragraph of the discussion. We would also like to acknowledge a mistake in the original manuscript, the model was trained across two, not four, GPUs and so the total number of training iterations was 792 and took 1hr and 21 minutes on two NVIDIA A100 48GB GPUs. We have added these details to the section "Cascade Mask R-CNN model training".

- It is unusual to train on such large images, did you experiment with e.g. resizing the input to much smaller (images of <1000 are common, and <500 very common in studies using Mask-RCNN). The GPU resource required to train at this size is substantial, it would be interesting to know how much quality drops off if the images are smaller.

The main issue that we found with downscaling is losing the resolution necessary to measure the silique traits, especially diameter. At 100% resolution, silique width is about 10 pixels across so reducing resolution would result in relatively large losses that would remove much of the variation in this metric. A reasonably accurate estimation of phenotypic variation is needed for the genetic mapping. We did

undertake pilot experiments with downscaled images with the same training parameters. For example, an image size of 2040x1440 (40% scaling and therefore still quite high resolution) leads to the AP_0.5:0.95 metric dropping down to 78.8 (box) and 38.4 (segm). We saw a much more drastic drop in the AP_0.75 (segm) to 20.0. This lower AP is most likely caused by the extremely long shapes of the siliques and, when resizing occurs to calculate the IoU metrics against the ground-truth data, this extreme shape and small size results in poor alignment between the two masks ultimately leading to a poor IoU score. Therefore, while the qualitative results of the downscaled images seemed largely acceptable, the lower resolution results in the loss of fine detail of these very small objects within these images. This leads to poorer phenotypic data that does not have the resolution necessary for QTL analysis.

Moreover, while alternative solutions, such as dividing images into smaller patches, could preserve resolution and detail for siliques, they introduce complexity in splitting and reassembling, particularly for future studies focusing on positional branch-level traits. Thus, we chose a higher resolution (compared to the popular setting of less than 1000 × 1000) that balances image quality with the capabilities of our available GPU resources.

To clarify, we have also now included the model of GPU used for training.

- The way silique volume is calculated is not absolutely clear to me as written. I think you are using the distance transform at each point along the skeleton to estimate diameter, and then simulating a cylinder where the "height" is oriented on the XY plane? If so, perhaps add a qualification here about the orientation of these cylinders to make this slightly more clear.

Thank you. To clarify, the silique volume was calculated as a series of 1-voxel high cylinders whose height is oriented along the spinal line, at every single pixel of the centre length line (spinal line). We have updated the manuscript as follows:

"The silique volume is based on the Euclidean distance transform values computed from the silique mask. For each point along the central length line (spinal line) of the silique, the distance transform is calculated as the distance to the nearest border of the mask along a line orthogonal to the length line. These values serve as the radius, r, of a series of 1-voxel high cylinders, with a volume of πr^2h, where $h = 1$, centred on each point of the spinal line. Summing the volumes of all cylinders along the spinal line approximates the total silique volume, assuming a cylinder-like morphology where each cross-section is circular with a radius equal to the distance transform."

- Your segmentation results in Figure 2 seem qualitatively very good, while your accuracy metrics seem to be around 55% for AP. This is quite a bit lower than the detection accuracy of 88%. What accounts for this drop in accuracy? Are the borders sometimes a bit too big or too small? How does this affect the individual phenotypic traits you've measured?

We believe the lower AP_0.5:0.95 segmentation metric is due to the shape and size of the silique being detected. Firstly, the AP_0.5 metric was 94% and AP_0.75 66%, meaning that most siliques are achieving an IoU value between these two thresholds. SIliques are very long and thin, with a large circumference-to-area ratio, whilst also being quite small within the images (having a typical area of around 900 pixels, but being only 10 pixels in diameter). These extreme values mean that small changes in the predicted mask, being larger or smaller, might drastically change the calculated IoU value, lowering the calculated accuracy. However, if the masks follow the shape of the silique then the length and diameter metrics won't be largely affected

We noticed that downsized images can lead to other issues. For example, within these images there are many overlaps between siliques, where the box detection is capable of recognising each instance, but the mask generation branch gets confused as to which silique needs to be segmented and therefore can fail. This rarely happens when the occlusion is caused by siliques meeting perpendicularly. But when siliques are nearly parallel and in close proximity, the model may fail, leading to inaccurate measurements. This may be a function of the underlying (Cascade) Mask R-CNN architecture and the way it crops the feature map to produce masks: when siliques are in close proximity, large regions of the feature map containing the other silique are included leading to super-sized aberrant measurements.

Switching a different instance segmentation architecture may resolve this specific issue, however our filtering system typically removes these aberrant masks from the analysis (and, as we discuss in the Discussion, this only occurs in on average 7.3% of siliques.)

Reviewer #2: In this paper, the authors developed a pipeline to phenotype Arabidopsis fruit morphological traits using computer visions and to perform QTL analysis on the traits. The authors achieved good phenotyping accuracy and found QTL that are associated with the traits. This study provides insights into phenotyping using machine learning models as a cheap and high-throughput method. There are a couple of minor issues that I think worth clarifying:

**   I suggest that the authors re-organize or re-write the first section of the introduction. It should introduce why image-based plant phenotyping is needed. What is the problem of conventional phenotyping?*

We have included a paragraph in the introduction that goes into more detail as to the background of high-throughput phenotyping of Arabidopsis thaliana, and the application of computer vision/RGB images. Your comment motivated us to discuss the potential future value of immortalising these types of datasets through image analysis. We would hope that others will be enabled to extract other types of phenotypic data on traits, such as silique spacing and orientation, that require other approaches. Thus, the images should allow for analysis beyond the aims of the original experiment.

The topic of how fruit sizes and shapes are affected by pollination can be better integrated into the introduction.

Thank you for highlighting the role of pollination in influencing pod size. Pollination is indeed a key factor in determining reproductive success in many species including Arabidopsis, as it directly affects subsequent seed development, which is in turn critical for pod growth and final size. (*Vivian-Smith A., Luo M., Chaudhury A., Koltunow A. Fruit development is actively restricted in the absence of fertilization in Arabidopsis. Development. 2001;1284(1):2321–2331. doi: 10.1242/dev.128.12.2321)*. Even though pollination isn't the main focus of our studies, it is definitely a likely contributory factor in determining fruit size and we appreciate this opportunity to contextualize our work. We have therefore incorporated an additional reference and discussions as needed to further clarify the connection between pollination dynamics and pod size in the final manuscript.

* *In page 3 under Image annotation using the GIMP image annotator, 55 images were used for training and testing. Were the training and testing on the annotation of the images instead of phenotyping? Why only used 55 when there were 7132 images after filtering? What was the accuracy of annotating using GIMP? Were the 7132 images all annotated using this method?*

Yes, the annotated images were used to train the Cascade Mask R-CNN model, with 80% used directly for training and 20% kept aside for evaluation of the results. We only annotated 55 images (but 2940 individual silique annotations within those images) - annotation is a very time-intensive task. The term 'annotation' refers to the *human* annotation of images for training/evaluation. We sped up the annotation process by using the GIA` tool we developed, which assists the user by using the classical computer vision tools inside GIMP. We did not directly measure any accuracy of the

outputs generated by GIMP as it only assists the user by speeding up annotation and does not perform annotations itself. The user has the final say on whether to adjust annotations in GIMP. We have included an example animation demonstrating the annotation process using the tool on the GitHub repository (https://www.github.com/kieranatkins/gimp-image-annotator). 55 images were annotated by hand, 44 for training and 11 for testing. Once trained, the model generated outputs on the full 7132 images.

*

1. Authors could report how many samples are removed in the first and second steps respectively in the two-step filtering process. About ¼ of the original pots were removed. It doesn't seem like a good phenotyping tool if most of them are removed in the second stage.

For clarity, we have also removed reference to 'two-step' filtering, in favour of 'two-level' filtering, to ensure the reader knows that the filtering is not required to be sequential and that the two levels are independent.

There were indeed two levels of filtering. One, the pot-level filtering, was based on biological criteria (as per the original experiment by Biernaskie & Garzón-Martínez et al.) and is not associated with the image analysis and extraction of phenotypic measurements. In brief, the size of the biological experiment meant that some plants did not grow well so, following the necessary biological experimental design criteria, replicates of that genotype would be removed from the QTL analysis. The Mask R-CNN model, however, generated outputs for all images within the dataset. The second, the silique-level filtering was employed because some outputs of the model were imprecise, and as mentioned in the discussion, this only occurs for, on average, 7.3% of outputted masks (siliques) but this did not remove any biological samples from the analysis. We have revised the relevant paragraph to provide better clarity.We have included the line "702 samples were therefore removed from the QTL analysis." in section "Phenotypic metrics at the sample level". Also, for reference, the file we include in the repository (https://github.com/kieranatkins/silique-detector/blob/main/qtl_analysis/data/phenotype_out_7934899.csv) contains the direct outputs of the network over every single image.

To reiterate, the first filtering level excludes aberrant samples based on the original experimental protocol; this sample removal is independent of the phenotyping tool used. The second level ensures the quality of the phenotype data extracted at the silique level, with only 7.3% of siliques excluded on average during this phase, many

due to occlusions. This minimal exclusion does not significantly affect our phenotypic metrics, as we have focused on percentiles, which are generally robust against outliers.

2. Why was it important to ensure each mask represented a single connected shape? Shouldn't a good phenotyping tool recognize overlapping shapes?

The instance segmentation model produces an individual mask for each detected silique, not an overall semantic segmentation output with a single mask denoting which pixels within the image are "silique". The model produces a mask (amodally) for each silique within the image, denoting which pixels belong to that instance of silique, irrespective of overlap.

* *How was fruit size measured? Was silique area the same as fruit size? Or does it mean percentiles on all the traits (length, area, etc.)? If it is the case, then it's better to write traits instead of fruit size.*

We originally used the term "fruit size" to generically refer to any of the fruit morphology traits collected. We have checked all occurrences of this term and updated them for clarity when referring to the specific metrics we have collected.

* *A zoom-in picture of Figure 2 might be better. It is hard to see red masks inside of boxes.*

Thank you - We have included zoomed cutouts of each of the images in Figure 2.

* *In the last paragraph of the discussion, the authors mentioned that only 44 images were used. Were the 44 images part of the 55 annotated images? Why were only 44 images used in the training set? Would it not be better to train the models with more images?*

While only 44 images were used in training, this represents 2282 independent instances. These 44 were the 80% training split of the 55-image annotated dataset. We found that these 44 annotated images successfully trained the model to produce satisfactory results.

As discussed in the last paragraph of the discussion, these images typically contain many individual silique annotations, and we believe this high quantity of annotations produces highly informative loss values during training, to require less images. This is backed up by the qualitative outputs we show, the AP metrics and the successful relation to the genome through QTL analysis.

* *I recommend that the authors add a section in methods and material about filtering images, testing and training data set, and cross-validation. How many images and samples were filtered or excluded in each step, and what was the reason for each filtering step?*

As mentioned above, the only sample-level filtering that occurred was due to the original experimental design for QTL mapping comparing plants under or not under competition, and not required for the function of model per se. Please see the following revised paragraph, which we hope provides better clarity.

" Prior to QTL analysis, we employed a two-level filtering process to ensure that the extracted phenotypic data were biologically relevant. The first level was applied to the pots (i.e., samples), excluding aberrant samples unrelated to the quality of the model predictions. The second level was applied to the siliques, removing model-detected siliques based on the quality of the generated masks to improve the accuracy of the extracted traits."

It seemed wasteful when over 7000 images were taken but only 44 images were used in the deep learning. And how many permutations have been done on the 5-fold cross-validation for instance segmentation evaluation?

Thank you for your question regarding the number of examples used for training and the cross-validation methods. We have clarified this in the revision by adding a paragraph in the section titled 'Model Evaluation for Instance Segmentation'. The annotated dataset was divided into 44 images for training (containing a total of 2,282 individual silique annotations) and 11 images for testing (with 652 silique annotations). The model trained with these 44 images achieved satisfactory performance, as evaluated through instance segmentation metrics on the annotated test set of 11 images, as well as through qualitative assessment via visual inspection. Ultimately, the model can be further tested on the entire experimental dataset of over 7000 images to verify its performance through QTL analysis.

We did not perform 5-fold cross validation for the instance segmentation model. K-fold cross-validation is computationally expensive; thus, we validate the model via the more popular hold-out cross-validation methods for deep learning models. The model was trained on 80% of the annotated images and tested on the remaining 20%. However, the majority of the images do not contain labels for instance segmentation, limiting our ability to evaluate the model using instance segmentation metrics. The QTL analysis also exists as a form of verification that the model is performant enough, from those 44 images, to produce precise-enough phenotype data that can be related to the genome. We judged these two evaluation systems to evaluate the outputs of the model, both through the outputted masks and the derived phenotype data, satisfactorily.
