# Supplementary material for: Unlocking the power of AI for phenotyping fruit morphology in Arabidopsis
Source: Gigascience. 2025 Feb 12;14:giae123. doi: 10.1093/gigascience/giae123 (PMC11816797; doi:10.1093/gigascience/giae123)
Supplement: giae123_Response_to_Reviewer_Comments_Original_Submission [file giae123_response_to_reviewer_comments_original_submission.pdf]

Author's Response To Reviewer Comments

See attached response letter.
