# Supplementary material for: Unlocking the power of AI for phenotyping fruit morphology in Arabidopsis
Source: Gigascience. 2025 Feb 12;14:giae123. doi: 10.1093/gigascience/giae123 (PMC11816797; doi:10.1093/gigascience/giae123)
Supplement: giae123_Response_to_Reviewer_Comments_Revision_1 [file giae123_response_to_reviewer_comments_revision_1.pdf]

Author's Response To Reviewer Comments

Dear Dr Nicole Nogoy,

Thank you for your feedback and provisional acceptance of our manuscript.

We have made all the requested editorial changes, ensuring compliance with the software citation principles and properly referencing GitHub, Zenodo, and similar platforms.

Please let us know if further changes are needed.

Best wishes,

Kieran Atkins, John Doonan, and Chuan Lu
on behalf of the co-authors
