# Supplementary material for: Unlocking the power of AI for phenotyping fruit morphology in Arabidopsis
Source: Gigascience. 2025 Feb 12;14:giae123. doi: 10.1093/gigascience/giae123 (PMC11816797; doi:10.1093/gigascience/giae123)
Supplement: giae123_Reviewer_1_Report_Original_Submission — Michael Pound -- 11/24/2024 [file giae123_reviewer_1_report_original_submission.pdf]

Reviewer Report

Title: Unlocking the Power of AI for Phenotyping Fruit Morphology in Arabidopsis

Version: Original Submission **Date:** 11/24/2024

Reviewer name: Michael Pound

Reviewer Comments to Author:

The authors present a deep learning approach to the detection and segmentation of Arabidopsis fruit, along with a QTL study linking genotype to fruit phenotyping. The paper is well written and clear, and I believe represents a useful and interesting study in this area. I have some minor queries / suggestions:
- If I understand your experimental numbers correctly, you have produced a set of 55 annotated images, which are split 80:20 into training and testing. This would leave a testing set size of ~11 images, is this correct? It would be helpful to include the exact values in the manuscript. Similarly, you train for 36 epochs, why did you choose this value. If an epoch represents one look over the dataset, with a training set of ~44 images across 4 GPUs, you would require only about 400 training iterations to complete the entire training process, is this correct? This is likely fine, but an estimate of the total training time would be helpful.
- It is unusual to train on such large images, did you experiment with e.g. resizing the input to much smaller (images of <1000 are common, and <500 very common in studies using Mask-RCNN). The GPU resource required to train at this size is substantial, it would be interesting to know how much quality drops off if the images are smaller.
- The way silique volume is calculated is not absolutely clear to me as written. I think you are using the distance transform at each point along the skeleton to estimate diameter, and then simulating a cylinder where the "height" is oriented on the XY plane? If so, perhaps add a qualification here about the orientation of these cylinders to make this slightly more clear.
- Your segmentation results in Figure 2 seem qualitatively very good, while your accuracy metrics seem to be around 55% for AP. This is quite a bit lower than the detection accuracy of 88%. What accounts for this drop in accuracy? Are the borders sometimes a bit too big or too small? How does this affect the individual phenotypic traits you've measured?

Methods

Are the methods appropriate to the aims of the study, are they well described, and are necessary controls included? Choose an item.

Conclusions

Are the conclusions adequately supported by the data shown? Choose an item.

Reporting Standards

Does the manuscript adhere to the journal's guidelines on minimum standards of reporting? Choose an item.

Choose an item.

Statistics

Are you able to assess all statistics in the manuscript, including the appropriateness of statistical tests used? Choose an item.

Quality of Written English

Please indicate the quality of language in the manuscript: Choose an item.

Declaration of Competing Interests

Please complete a declaration of competing interests, considering the following questions:

- Have you in the past five years received reimbursements, fees, funding, or salary from an organisation that may in any way gain or lose financially from the publication of this manuscript, either now or in the future?
- Do you hold any stocks or shares in an organisation that may in any way gain or lose financially from the publication of this manuscript, either now or in the future?
- Do you hold or are you currently applying for any patents relating to the content of the manuscript?
- Have you received reimbursements, fees, funding, or salary from an organization that holds or has applied for patents relating to the content of the manuscript?
- Do you have any other financial competing interests?
- Do you have any non-financial competing interests in relation to this paper?

If you can answer no to all of the above, write 'I declare that I have no competing interests' below. If your reply is yes to any, please give details below.

I declare that I have no competing interests

I agree to the open peer review policy of the journal. I understand that my name will be included on my report to the authors and, if the manuscript is accepted for publication, my named report including any attachments I upload will be posted on the website along with the authors' responses. I agree for my report to be made available under an Open Access Creative Commons CC-BY license (http://creativecommons.org/licenses/by/4.0/). I understand that any comments which I do not wish to be included in my named report can be included as confidential comments to the editors, which will not be published.

Choose an item.

To further support our reviewers, we have joined with Publons, where you can gain additional credit to further highlight your hard work (see: https://publons.com/journal/530/gigascience). On publication of this paper, your review will be automatically added to Publons, you can then choose whether or not to claim your Publons credit. I understand this statement.

Yes Choose an item.
