# Supplementary material for: Unlocking the power of AI for phenotyping fruit morphology in Arabidopsis
Source: Gigascience. 2025 Feb 12;14:giae123. doi: 10.1093/gigascience/giae123 (PMC11816797; doi:10.1093/gigascience/giae123)
Supplement: giae123_Reviewer_2_Report_Original_Submission — Fangyi Wang -- 12/3/2024 [file giae123_reviewer_2_report_original_submission.pdf]

Reviewer Report

Title: Unlocking the Power of AI for Phenotyping Fruit Morphology in Arabidopsis

Version: Original Submission **Date:** 12/3/2024

Reviewer name: Fangyi Wang

Reviewer Comments to Author:

In this paper, the authors developed a pipeline to phenotype Arabidopsis fruit morphological traits using computer visions and to perform QTL analysis on the traits. The authors achieved good phenotyping accuracy and found QTL that are associated with the traits. This study provides insights into phenotyping using machine learning models as a cheap and high-throughput method. There are a couple of minor issues that I think worth clarifying:
* I suggest that the authors re-organize or re-write the first section of the introduction. It should introduce why image-based plant phenotyping is needed. What is the problem of conventional phenotyping? The topic of how fruit sizes and shapes are affected by pollination can be better integrated into the introduction.
* In page 3 under Image annotation using the GIMP image annotator, 55 images were used for training and testing. Were the training and testing on the annotation of the images instead of phenotyping? Why only used 55 when there were 7132 images after filtering? What was the accuracy of annotating using GIMP? Were the 7132 images all annotated using this method?
* Authors could report how many samples are removed in the first and second steps respectively in the two-step filtering process. About Â¼ of the original pots were removed. It doesn't seem like a good phenotyping tool if most of them are removed in the second stage. Why was it important to ensure each mask represented a single connected shape? Shouldn't a good phenotyping tool recognize overlapping shapes?
* How was fruit size measured? Was silique area the same as fruit size? Or does it mean percentiles on all the traits (length, area, etc.)? If it is the case, then it's better to write traits instead of fruit size.
* A zoom-in picture of Figure 2 might be better. It is hard to see red masks inside of boxes.
* In the last paragraph of the discussion, the authors mentioned that only 44 images were used. Were the 44 images part of the 55 annotated images? Why were only 44 images used in the training set? Would it not be better to train the models with more images?
* I recommend that the authors add a section in methods and material about filtering images, testing and training data set, and cross-validation. How many images and samples were filtered or excluded in each step, and what was the reason for each filtering step? It seemed wasteful when over 7000 images were taken but only 44 images were used in the deep learning. And how many permutations have been done on the 5-fold cross-validation for instatnce segmentation evaluation?

Methods

Are the methods appropriate to the aims of the study, are they well described, and are necessary controls included? Choose an item.

Conclusions

Are the conclusions adequately supported by the data shown? Choose an item.

Reporting Standards

Does the manuscript adhere to the journal's guidelines on minimum standards of reporting? Choose an item.

Choose an item.

Statistics

Are you able to assess all statistics in the manuscript, including the appropriateness of statistical tests used? Choose an item.

Quality of Written English

Please indicate the quality of language in the manuscript: Choose an item.

Declaration of Competing Interests

Please complete a declaration of competing interests, considering the following questions:

- Have you in the past five years received reimbursements, fees, funding, or salary from an organisation that may in any way gain or lose financially from the publication of this manuscript, either now or in the future?
- Do you hold any stocks or shares in an organisation that may in any way gain or lose financially from the publication of this manuscript, either now or in the future?
- Do you hold or are you currently applying for any patents relating to the content of the manuscript?
- Have you received reimbursements, fees, funding, or salary from an organization that holds or has applied for patents relating to the content of the manuscript?
- Do you have any other financial competing interests?
- Do you have any non-financial competing interests in relation to this paper?

If you can answer no to all of the above, write 'I declare that I have no competing interests' below. If your reply is yes to any, please give details below.

I declare that I have no competing interests

I agree to the open peer review policy of the journal. I understand that my name will be included on my report to the authors and, if the manuscript is accepted for publication, my named report including any attachments I upload will be posted on the website along with the authors' responses. I agree for my

report to be made available under an Open Access Creative Commons CC-BY license (http://creativecommons.org/licenses/by/4.0/). I understand that any comments which I do not wish to be included in my named report can be included as confidential comments to the editors, which will not be published.

Choose an item.

To further support our reviewers, we have joined with Publons, where you can gain additional credit to further highlight your hard work (see: https://publons.com/journal/530/gigascience). On publication of this paper, your review will be automatically added to Publons, you can then choose whether or not to claim your Publons credit. I understand this statement.

Yes Choose an item.
